# Fire-resistant polymer composites based on mineral fillers

**DOI:** 10.1039/d5ra09525e

**Published:** 2026-01-29

**Authors:** F. Mustafayeva, N. Kakhramanov, Kh. Allahverdiyeva, T. Babayeva, N. Ashurova

**Affiliations:** a Institute of Polymer Materials of Ministry of Science and Education Republic of Azerbaijan Sumgait City AZ5004 Azerbaijan mustafayevafatima@mail.ru fatime.mustafayeva@sdu.edu.az; b Sumgait State University Sumgait City AZ5008 Azerbaijan

## Abstract

This review examines the role of mineral fillers in improving the fire resistance property of polymer composites during combustion. The mechanism of action of mineral filler particles and the nature of changes in the fire resistance of composites depending on the ratio of components in the mixture are considered. The separate and combined effects of natural and modified mineral fillers on the heat resistance and fire resistance of polymer composites are also considered. Mineral fillers such as zeolite, huntite, hydromagnesite, sepiolite, basalt, vermiculite, montmorillonite, perlite, mica, talc, halloysite, kaolin and wollastonite are used to evaluate the flame-retardant properties of composites. Results from different studies on the influence of various types of mineral flame retardants, structural features of the polymer matrix and the composition of composites on the flame-retardant mechanism are systematically evaluated. In conclusion, the promising possibilities of using mineral flame retardants in various fire-resistant polymer composites are considered.

## Abstract 2

This review examines the role of mineral fillers in improving the fire resistance property of polymer composites during combustion.

## Graphical abstract

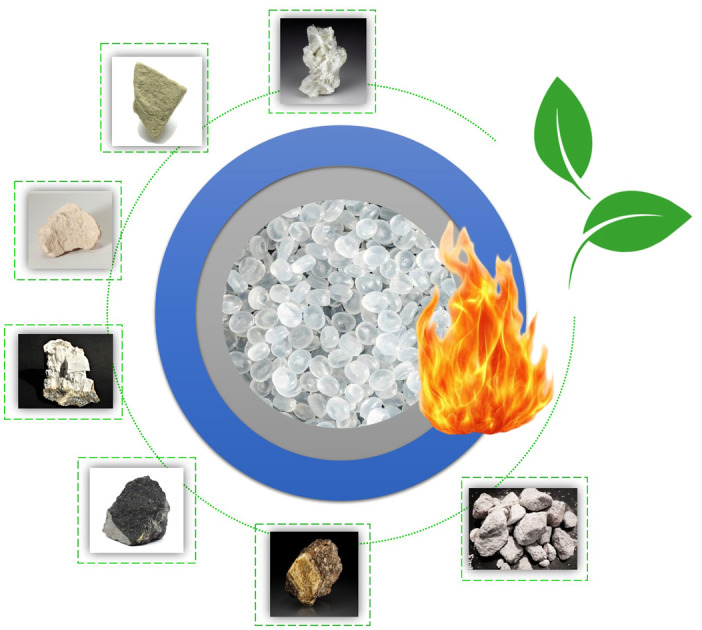

## 1. Introduction

Most polymers are highly flammable, which is one of the main reasons for their limited use as a polymer matrix.^1^ Burning of polymer materials result in the release of smoke and toxic gases, which are hazardous to human health. Consequently, reducing their flammability is necessary for the utilization of polymer matrix composites.^2–4^ There are several known ways to improve the fire resistance property of polymer materials.^5^ For example, the introduction of flame retardants is the simplest and most effective solution, which makes it possible not only to reduce the flammability of the polymer matrix but also improve its physical, mechanical and thermal characteristics. Additionally, as shown in the results of practical studies, fire-resistant composites can be processed using high-performance processing methods such as extrusion and injection molding. The main function of fire retardants is to slow down the combustion process and limit the spread of flames when a material is exposed to flames.^6^ A large variety of compounds, from inorganic to organic molecules, are used as flame retardants, synergists and smoke suppressants.^7–12^

As a rule, flame retardants act either in the gas or condensed phase through various chemical and physical mechanisms that prevent the combustion process: they change the course of pyrolysis by reducing the amount of combustible gases or volatile low-molecular products released. As a result, during combustion, a less flammable and carbonized coating is formed, acting as a barrier between the material and the flame, stopping the oxygen access; suppressing the spread of fire due to the deactivation of free radicals produced by the halogen derivatives of the flame retardant; and reducing the amount of heat released by the flame, thereby preventing subsequent high-temperature pyrolysis reactions.^13^

In recent years, the demand by researchers and manufacturers for flame retardants based on mineral fillers has increased because of their obvious fire-resistant properties.^14^ Mineral flame retardants help minimize toxic emissions and smoke and suppress the generation of corrosive gases during a fire. In this regard, there was a need to search for environmentally friendly substitutes for halogenated flame retardants. Taking into account the above-mentioned facts, this review attempts to categorize research in the field of fire-resistant polymer composites depending on the type of polymer matrix and various mineral flame retardants. Presently, there is a need to develop scientific approaches to study the influence of each component in polymer composites on the nature of the change in the combustion process mechanism of the polymer matrix. To achieve this, it is necessary to consider the fundamental scientifically based principles and approaches for controlling the combustion process of fire-resistant composites and study the mechanism of thermal decomposition by selecting and changing the ratio of flame retardants in combination with natural minerals, compatibilizers and modifying additives. This is a rather complex process, which requires generalization and research into the cause-and-effect processes and phenomena that contribute to the formation of fire-resistant structural materials.

In this regard, the aim of this work is to show the advantageous features of the separate and combined influence of various natural mineral flame retardants on the combustion mechanism and the development of a concept to obtain fire-retardant polymer composites. For this purpose, herein, we present a critical review of the research in this field in the last 10 years.

## 2. Flame retardancy test methods

Currently, the effectiveness of flame retardants is assessed by comparing data obtained in determining the flammability, fire resistance and fire hazard of various materials that contain flame retardants. The methods for determining the flammability of these materials can be divided into four groups: (1) kinetic, (2) thermal, (3) temperature-based, and (4) concentration-based methods.

The performance of a flame retardant can show significant discrepancies from one test to another, as the conditions under which tests are conducted and the criteria for success or failure are highly variable.^15^ A set of test methods is typically employed to comprehensively and deeply assess the flame-retardant performance of polymer composites.

### 2.1. Limiting oxygen index (LOI)

The lowest concentration of oxygen necessary for a material to sustain flaming combustion in a blend of oxygen and nitrogen is known as the LOI. It is standardized in the United States (ASTM D 2863) and in France (NF T 51-071), as well as internationally (ISO 4589).^16^ In the plastic industry, this method remains an indispensable screening and quality control tool, as it effectively assesses both the ignitability and flammability resistance of a material. An LOI of 21% has significant practical significance. Given that the oxygen concentration in the atmosphere is approximately 21% by volume, materials with an LOI below this value exhibit increased flammability. Conversely, materials with an LOI above 21% are resistant to combustion under standard conditions and require an external energy source to burn. Materials are classified by their LOI as follows: flammable, with an LOI less than 20.95%, slow-burning, with an LOI ranging from 20.95% to 28%, self-extinguishing, with an LOI from 28% to 100%, and intrinsically non-flammable, with an LOI exceeding 100%.^17^

### 2.2. Cone calorimetry (CC)

CC has been proven to be a reliable method for quantifying the combustion properties of materials under fire, contributing to its growing popularity. Cone calorimetry is one of the most effective medium-sized polymer fire behavior tests. Following the standards of ASTM E1354 or ISO 5660, a cone calorimeter can deliver an in-depth understanding of the flammability of polymers in the fields of fire engineering and science.^18^ Cone calorimetry provides information on the heat release rate (HRR), mass loss rate (MLR), total heat release (THR), time to ignition (TTI), time to flame out (TFO), levels of oxygen, carbon monoxide, and carbon dioxide, and total smoke released (TSR).^16^

### 2.3. Underwriters laboratories 94 (UL94)

Published by Underwriters Laboratories, the UL-94 flammability test is the most common protocol for assessing the flame resistance of plastic materials.^19^ The UL-94V (vertical burning test) assessment for vertical samples remains the standard in the polymer sector to gauge flammability. This evaluation provides material classifications of V-0, V-1, V-2, and NR (not rated). A substance that fails to satisfy the requirements of the Vertical Burning Test may be examined according to UL-94HB, a Horizontal Burn Test, and typically earns an HB-rating.

## 3. Flame-retardant polymer composites based on mineral fillers

Mineral filler flame retardants are one of the most important classes of flame retardants used in the composition of polymer matrices.^20–22^ They can endow polymer composites with structural strength and reinforcement, in addition to reducing their flammability to acceptable levels. Heat capacity of the filler, decomposition endotherm, heat capacity of the gas or vapor, and heat capacity of the residue are the four flame retardant effects that have been measured. The structure and composition (Table 1) of minerals determine how they interact with the polymer matrix, providing a synergistic effect and improved thermal stability. A viable substitute is provided by naturally occurring flame-retardant minerals, which are made up of non-metallic atoms such as silicon, phosphorus, oxygen, and carbon as well as metallic elements such as aluminum, magnesium, calcium, iron, and titanium.^23^ Additionally, as will be shown below, these minerals have exceptional flame-retardant properties due to the synergistic effect among different types of elements.

Chemical composition of raw minerals

**Table:** 

Vermiculite^24^									
Oxides	SiO_2_	MgO	Al_2_O_3_	K_2_O	Fe_2_O_3_	TiO_2_	CaO	Na_2_O	Loss on ignition
Percentage	36.3	14.6	14.8	4.8	12.4	2.7	4.2	0.3	9.10

**Table:** 

Montmorillonite^25^									
Oxides	SiO_2_	Al_2_O_3_	Fe_2_O_3_	FeO	CaO	MgO	Na_2_O	Trace	Loss on ignition
Percentage	63.02	21.08	3.25	0.35	0.65	2.67	2.57	0.72	5.64

**Table:** 

Perlite^26^									
Oxides	SiO_2_	Al_2_O_3_	Fe_2_O_3_	TiO_2_	CaO	MgO	K_2_O	Na_2_O	Loss on ignition
Percentage	72.48	13.15	1.23	0.15	1.13	0.35	4.21	3.56	3.54

**Table:** 

Kaolin^27^										
Oxides	SiO_2_	Al_2_O_3_	Fe_2_O_3_	MnO	MgO	CaO	K_2_O	TiO_2_	P_2_O_5_	Loss on ignition
Percentage	56.84	30.70	0.89	0.01	0.16	0.05	1.53	0.27	0.04	9.85

**Table:** 

Wollastonite, determined by X-ray fluorescence^28^						
Oxides	CaO	SiO_2_	Al_2_O_3_	MgO	Fe_2_O_3_	K_2_O
Percentage	51.2 ± 0.6	46.4 ± 0.3	0.79 ± 0.04	0.70 ± 0.05	0.52 ± 0.06	0.14 ± 0.02

A novel type of composite material, natural mineral-reinforced polymers, is becoming increasingly significant in a number of industrial areas.^29^ Polymers reinforced with natural minerals are composites in which they act as reinforcing elements that improve the mechanical and thermal properties of the polymer matrix.^30^ Currently, mineral fillers are considered cost-effective and efficient additives for the production of various halogen-free flame-retardant materials. Their behavior is characterized by increased thermal stability, non-volatility and long service life. It should be noted that to impart the required degree of fire resistance to polymers, it is necessary to use a significant amount of filler, which in some cases can lead to the deterioration of the physical and mechanical properties of the composite. Therefore, the correct choice of a mineral flame retardant with the minimum permissible content in the polymer matrix is an important, but at the same time complex scientific and technical problem. For this purpose, a whole range of mineral fillers of various origins has been investigated for their applicability as flame retardants in polymer composites. The availability and large deposits of mineral deposits allow for the expansion of their practical use as an environmentally friendly source of polymer fillers. It is quite clear that the use of minerals alone is not sufficient to achieve an acceptable level of fire resistance in a polymer composite. However, owing to the creation of a protective surface barrier, it was discovered that various compositions containing flame retardants and mineral fillers were effective in lowering the rate of heat release.

For a comprehensive analysis of the role of mineral flame retardants in influencing the fire protection properties of polymer composites, it seems interesting to consider the separate influence of different types of mineral fillers on their fire resistance.

### 3.1. Flame-retardant polymer composites based on zeolites

Zeolites are aluminosilicate minerals with a three-dimensional structure composed of interconnected SiO_4_ and AlO_4_ tetrahedra.^31^ Zeolites have the general chemical formula *M*_*a*/*x*_[(AlO_2_)_*a*_·(SiO_2_)_*b*_]·*z*H_2_O, where *z* is the number of water molecules per unit cell, *a* and *b* are the total number of tetrahedral of Al and Si per unit cell, respectively, with a ratio *b*/*a* ≥1 and *M* represents any alkali or alkaline earth metal atom needed to balance the charge. Zeolites possess the characteristics of large internal and external surface areas, distinctive nanoscale pores, great thermal stability, environmental friendliness, and a high capacity for cation exchange. Thus, zeolites are a type of potential aluminosilicate filler that could have a synergistic impact in flame-retardant formulations.^32^ According to reports, adding zeolite to thermoplastic polymers can both expedite their flame-retardancy performance and minimize the amount of flame retardant required without compromising their flame-retardant behavior.

**Fig. 1:**
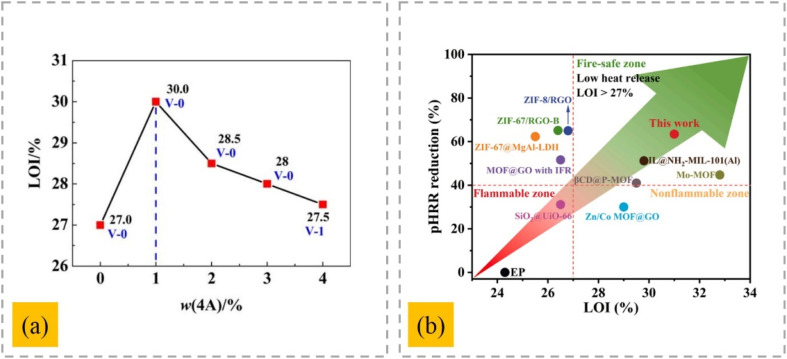
(a) Effect of 4A zeolite on the flame retardancy of the EVA/IFR composites,^33^ reproduced from ref. 33 with permission from John Wiley and Sons, Copyright 2016. (b) Comparison of the fire resistance property of the EVA/IFR composites with that of the previously reported zeolite- and metal–organic framework-based flame retardants for EP,^37^ reproduced from ref. 37 with permission from Elsevier, Copyright 2022.

The thermal degradation, flame retardancy, and char formation of high-density polyethylene (HDPE) composites were examined in relation to the synthetic 4A zeolite and an intumescent flame-retardant (IFR) system made up of ammonium polyphosphate (APP) and tris(2-hydroxyethyl)isocyanurate (THEIC).^31^ With an optimum content of 0.5 wt% zeolite 4 A and 25 wt% total flame retardant, the LOI value of the HDPE composite reached 26.3%. A low 4A zeolite loading can enhance the bench-scale combustion performance and encourage the production of more compact, highly graphitic char residue. The synergistic effects of 4A zeolite on an ethylene–vinyl acetate copolymer composite (EVA/IFR) were investigated. It was discovered that a tiny quantity of 4A significantly increased the LOI value of the EVA/IFR composite (Fig. 1a) and reinforced its flame-retardant performance by significantly lowering the combustion characteristics of the system.^33^ Consequently, all the composites passed the UL-94 V-0 rating test. When 4A was added to the EVA/IFR system, the phosphorus concentration in the char residue increased and a more graphitic structure was created.

Polyvinyl chloride/4A zeolite/urea cyanurate composites (P/UCA-Zlt) were designed and prepared *via* a melt intercalation method.^34^ Results showed that the composites based on UCA-Zlt exhibited better flame retardancy, better HCl scavenger properties, improved mechanical properties, and reduced level of degradation compared with pure polyvinyl chloride (PVC) upon the optimum modification of UCA (3 wt%)–Zlt (1 wt%). Thermogravimetric analysis (TGA) indicated that the stability of PVC against heat influence increased by adding UCA–Zlt, which was evident by the increase in its thermal decomposition temperature. According to the CC results for the PVC composites, their flame-retardant properties were enhanced. 4A zeolites and 4A zeolites containing La (4A-La) were incorporated into a polypropylene (PP) composite containing IFR.^35^ The flame retardancy of the PP composites greatly increased with the addition of both the 4A and 4A-La zeolites. The LOI for neat PP was only 18.1%; for PP/25 wt% IFR, it increased to 31.1%; and for PP/23.5 wt% IFR/1.5 wt% 4A or 4A-La, it further climbed to 34.1% and 35.5%, respectively. The peak heat release rate (pHRR) for PP is 1474 kW m^−2^, while that for PP/IFR, PP/IFR/4A, and PP/IFR/4A-La composite is 436, 299, and 248 kW m^−2^, respectively. It was proposed that the 4A-La zeolite might improve char production by the PP composites and change their thermal degradation characteristics. The impact of the 4A zeolite on the thermal stability and flame retardancy of aluminum β-(*p*-nitrobenzamide) ethyl methyl phosphinate (AlNP) in acrylonitrile–butadiene–styrene (ABS) copolymer was studied.^36^ ABS filled with 22 wt% AlNP and 3 wt% 4A zeolite achieved UL94 V-0 with an LOI value of 29.3% when the total loading of the additives was kept at 25 wt%. In contrast, ABS with 25 wt% AlNP alone only passed UL 94 V-1 with an LOI value of 26.0%. The CC results confirm that 4A zeolite can reduce the combustion heat and smoke output. Zhongwei Chen *et al.* mixed zeolites made from electrolytic manganese residue with 9,10-dihydro-9-oxa-10-phosphaphenanthrene-10-oxide and a ligand of a metal–organic framework as flame retardants (D-CoZ-BDC-1).^37^ The flame-retardant epoxy resin (EP) composite (EP/D-CoZ-BDC-1) exhibited an LOI of 31.0% (Fig. 1b). In comparison to EP, the pHRR, THR, peak smoke release rate, total smoke release, CO yield, and CO_2_ yield of EP/D-CoZ-BDC-1 decreased by 63.4%, 48.9%, 52.8%, 60.1%, 30%, and 48.7%, respectively. Excellent fire resistance was provided by the combined effects of pyrolysis in the creation of Co_3_O_4_, the phospholipid biphenyl structure, Al_2_O_3_ and SiO_2_ in the condensed phase, and PO˙ in the gas phase.

### 3.2. Flame-retardant polymer composites based on huntite & hydromagnesite

Huntite [Mg_3_Ca(CO_3_)_4_] and hydromagnesite [Mg_5_(CO_3_)_4_(OH)_2_·4H_2_O] are a natural blend and classified as carbonate minerals of the salt type.^38^ This natural mineral, due to its attractive properties, is mined and processed on an industrial scale as an alternative to widely used fire retardants, as it is characterized by low smoke generation, environmental safety, the absence of halogens, the possibility of recycling and low flammability. Hydromagnesite is also referred to as “magnesia white”.^39^ It is a rare natural hydrated alkaline magnesium carbonate mineral. Over the temperature range of 220 °C to 550 °C, it thermally breaks down in two phases, releasing carbon dioxide and water and leaving behind a solid residue of magnesium oxide. However, hydromagnesite contains impurities (CaCO_3_, Fe_2_O_3_, and SiO_2_) that might deteriorate the characteristics of polymeric materials.

When the flame retardancy mechanisms of inorganic minerals are generally examined, it is seen that these minerals, which consist of hydroxides or hydrous carbonates, decompose endothermically at temperatures between 200–400 °C.^40^ Water vapor and carbon dioxide produced by the decomposition reaction have a cooling effect on the surrounding environment, making it more difficult for the fire to spread further. Alternatively, the ceramic layer formed as a result of the reaction acts as a barrier between the flame and the flammable surface, blocking the contact of the surface with atmospheric oxygen. As a result, the spread of fire is prevented, thus protecting the polymeric materials from further exposure to flame and heat.

Studies on the synergistic effect of huntite & hydromagnesite (HH) in various polymer composites to increase their flame-retardant effect have led to their wider use. Boric acid and antimony oxide were used as auxiliary flame-retardant materials together with HH.^41^ The flame-retardant properties improved with an increasing mineral content and decreasing size. The flame-retardant qualities of the HH-reinforced polymeric composites significantly improved with an increase in the concentrations of boric acid and antimony oxide.

Acetyl tributyl citrate, a bio-based plasticizer, and HH, a natural flame-retardant component, were used to formulate ductile green flame-retardant poly(lactic acid) (PLA) composites.^42^ When 70% HH was added with 20% plasticizer, the greatest LOI value (36.2%) and UL-94 rating of V-0 were obtained. To enhance the flame-retardant performance of plasticized PLA, aluminum phytate (AlPt) was combined with HH.^43^ When 5 wt% AlPt was added to 55 wt% HH, the greatest LOI value and UL-94 rating of V-0 were obtained.

**Fig. 2:**
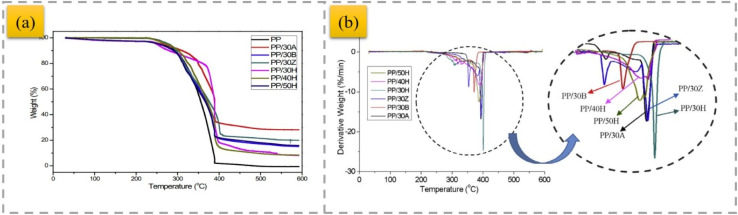
TGA (a) and derivative thermogravimetry (DTG) (b) curves of PP-based composites,^46^ reproduced from ref. 46 with permission from Elsevier, Copyright 2018.

Good synergistic effects were obtained using the HH mineral in combination with zinc borate (ZnB), resulting in an increase in the flame-retardant property of PP-based composites.^38^ Moreover, ZnB in combination with HH had a significant effect on the flammability characteristics of the composites, given that a significant increase in the formation of char was observed with the addition of ZnB. In addition, ZnB reduced the amount of smoke emitted during combustion of the composite. The combination of HH and phosphorus–nitrogen-based IFRs was investigated to improve the flame retardancy and maintain the flexibility of PP.^44^ As a result, composites with UL94 V0 flammability and 18% to 104% higher tensile strength than the equivalent HH-based flame retardants were obtained. Calcite and zeolite minerals, with well-known incombustibility properties, were used together with HH as auxiliary minerals to obtain better mechanical properties.^45^ A synergistic effect was obtained in flame-retardant PP containing HH by adding zeolite and calcite. The synergistic effect obtained by using zeolite with HH improved the deformation and strength characteristics of the composites. Nano-sized HH, antimony trioxide, bentonite and ZnB particles were employed to reinforce the PP matrix.^46^ TGA and flame-retardant tests showed that the decomposition temperature of the composites increased in the presence of HH compared to neat PP and PP composites containing other types of minerals. The results of this study show that an increasing HH concentration in PP composites leads to an increase in their thermal stability (Fig. 2).

The influence of microencapsulated red phosphorus (mRP) was examined on the flame-retardant, thermal, and mechanical characteristics of thermoplastic polyurethane (TPU) composites incorporating HH.^47^ The partial replacement of mRP with HH produced the greatest LOI value (32.5%), highest UL-94 rating (V-0), and lowest pHRR (155 kW m^−2^). The use of mRP resulted in a synergistic effect due to an increase in the barrier effect and the formation of residue in the condensed phase and active radicals in the gas phase. The effect of ZnB was studied on the flame-retardant and thermal properties of TPU containing HH.^48^ ZnB had no discernible impact on the flammability characteristics of the composites. Regardless of the content of ZnB added, the UL 94 rating remained the same, and the LOI value slightly increased at a ratio of 1 : 1. During the mass loss calorimetry investigations, the adjuvant impact of ZnB was seen to increase the generation of incombustible gases in the gas phase and the barrier effect of the residue in the condensed phase. The impact of expandable graphite (EG) on the flame-retardant qualities of TPU incorporating HH was investigated.^49^ The results of the flammability tests showed that HH and EG had a synergistic relationship. The greatest UL-94 rating of V-0 was found at ratios of 4 : 1, 3 : 2, and 1 : 1, whereas the largest LOI value was found at a ratio of 1 : 1 (HH : EG).

Flame-retardant PE matrix composites were prepared with hydromagnesite and Mg(OH)_2_ as flame retardants.^50^ The cost of the PE composite with a synergistic flame retardant is substantially less than that utilizing Mg(OH)_2_ as a flame retardant, assuming the same LOI and tensile strength. The synergistic flame retardant reduces the ineffective, even negative, decomposition of hydromagnesite, while maintaining an inhibitory effect on the high-temperature decomposition of the PE matrix in the presence of hydromagnesite. Simultaneously, a comparatively stable scaly protective layer is formed on the surface of the combustion region, and the synergistic flame composite loses more mass overall. The impact of calcium carbonate, magnesium carbonate, natural magnesium hydroxide, and HH mineral fillers on PE and EVA/PE flame-retardant polymer compounds was investigated in a different study.^51^ The fire-retardancy characteristics of the corresponding composites were found to be considerably changed by the mineral additives. The addition of EVA to the polymer composite formulation in the presence of various fillers improved its fire resistance. In a previous study,^52^ the aim was to the improve the thermal stability of polyethylene terephthalate by adding HH mineral. The obtained results proved that the composite system is more thermally stable than pure polyethylene terephthalate.

Table 2 presents some results on the influence of HH on the flammability of polymer composites.

**Table 2:** Fire properties of HH-based polymer composites

Formulation of composites		LOI	UL-94	pHRR (kW m^−2^)	Reference
Matrix	Additive				
PP	—	17.1	NR	—	46
	30 antimony trioxide	21.3	V-1		
	30 bentonite	19.2	V-2		
	30 zinc borate	20.5	V-1		
	50 huntite/hydromagnesite	25.5	V-0		
TPU	—	21.2	NR	668	47
	50 HH	25.7	V-1	290	
	7 mRP	23.2	V-2	474	
	43 HH + 7 mRP	32.5	V-0	155	
TPU	—	21.2	NR	668	48
	50 HH	25.7	V-1	290	
	4 HH + 1 ZnB	24.1	V-1	154	
	2 HH + 1 ZnB	24.0	V-1	147	
	1 HH + 1 ZnB	25.5	V-1	122	
	50 ZnB	26.8	V-1	103	
TPU	—	21.2	NR	—	49
	60 HH	31.2	V-0		
	20 EG	25.2	V-1		
	25 HH + 25 EG	32.6	V-0		
PE	HH	26.6	HB	—	51
EVA/PE	HH	30.0	HB	—	

### 3.3. Flame-retardant polymer composites based on sepiolite

**Fig. 3:**
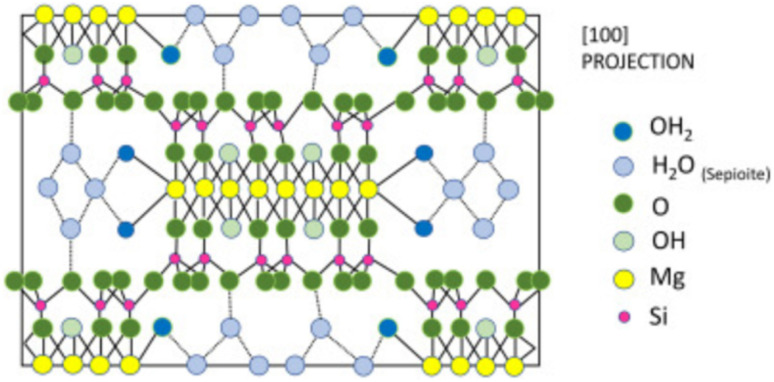
Schematic view of the structure of sepiolite,^54^ reproduced from ref. 54 with permission from Elsevier, Copyright 2022.

Sepiolite is a natural hydrated magnesium silicate that belongs to the 2 : 1 class of phyllosilicate minerals^53^ (Fig. 3). Its structure consists of two tetrahedral silica layers flanking a middle octahedral magnesium hydroxide-oxide layer. Sepiolite boasts a distinctive composition characterized by a fibrous, needle-like porous form featuring alternating tunnels and blocky formations aligned with the fiber axis. Sepiolite manifests a layered chain architecture with the chemical composition of Si_12_Mg_8_O_30_(OH)_4_(OH_2_)_48_H_2_O, which is commonly utilized as a synergistic agent in flame-retardant polymers owing to its effective catalytic properties. Consequently, research on the thermal stability of composites based on sepiolite and polymers has revealed its notable catalytic influence on polymer decomposition. It has been noted that the concentration of silica particles gathered on the surface of the composite, as well as their distribution across the exposed area during combustion, has a considerable impact on reducing the HRR. Sepiolite clay stands out as the most practical and accessible option due to its low cost, good availability, environmentally friendly nature, fibrous structure, and uninterrupted octahedral formations of [SiO_4_] and [MgO_6_], which makes it relatively suitable for the polymer matrix.^55^ The addition of sepiolite clay can certainly increase the flame retardancy of plastics. However, its modification can be beneficial in terms of better dispersion within the matrix.

The influence of sepiolite as a synergistic component on the flame retardancy and thermal degradation characteristics of composites based on polyamide 6,6 (PA66) and aluminium diethylphosphinate (AlPi) was studied.^56^ With a total content of 10 wt% AlPi + sepiolite mixture in the composite, the samples had UL-94 V-0 and LOI of 32.5%. Sepiolite facilitated the development of a char layer rich in flame-retardant elements on the surface of the materials, which improved the flame resistance of PA66.

In a previous study,^57^ K-SEP was obtained by modifying sepiolite with KH550, and two bio-based flame retardant and smoke suppression systems, AEGS and PEGS, were prepared by combining an APP IFR system (AEG) and piperazine pyrophosphate IFR system (PEG), respectively. The experimental results show that the LOI values of the composites with 3 phr K-SEP are 8.1% and 5.5% higher than that of the blank samples without sepiolite, respectively. The total smoke production (TSP) decreased by 26.3% and 14.3%, respectively, which proved that sepiolite has important research value in the field of flame-retardant and smoke suppression polymer materials. As is known, phosphorus-containing flame retardants are considered environmentally friendly and can be reinforced in polymers or functionalized on a polymer base to reduce their flammability. In a previous study,^58^ sepiolite fiber was primed with polydopamine. H_3_PO_3_ was grafted on polydopamine-coated sepiolite to prepare a robust filler to impart flame-retardant characteristics to flammable PP. Neat PP has an LOI of 17.5%, and the maximum improvement in the LOI was observed to be 30.2%.

### 3.4. Flame-retardant polymer composites based on basalt

Basalt fiber (BF) is considered an environmentally friendly, “green” and high-efficiency fiber with excellent characteristics, which is widely used in many fields.^59^ Basalts are formed by the rapid cooling of basaltic lava, equivalent to gabbro-norite magma, from the interior of the crust and exposed on or very close to the surface of the Earth.^60^ Basalts are composed of minute grains of plagioclase feldspar, pyroxene, olivine, biotite, hornblende, and less than 20% quartz. BFs have garnered significant attention, especially due to their impressive service temperature reaching 800 °C, melting range of 1500–1700 °C, remarkable fire barrier capabilities up to 1200 °C, and reasonable cost.^61^ Therefore, they are frequently utilized in the thermal insulation and passive fire protection fields. Furthermore, their capacity to maintain stability under high temperatures ensures that composites retain their performance features, even in extreme fire situations.^62^

**Fig. 4:**
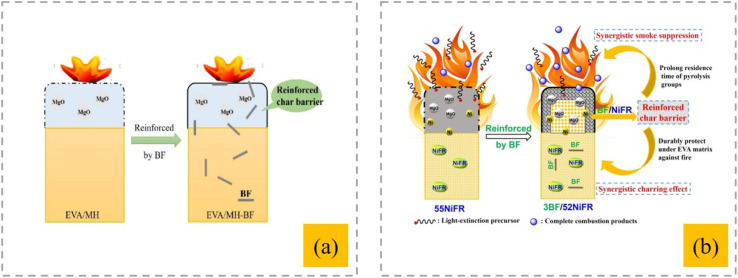
(a) Schematic of the combustion of EVA composites,^64^ this article is an open access article distributed under the terms and conditions of the Creative Commons CC BY license. (b) Synergetic flame-retardant mechanism,^65^ reproduced from ref. 65 with permission from John Wiley and Sons, Copyright 2020.

BF is inorganic in nature and therefore has a poor interfacial interaction with the polymer matrix. Generally, the use of surface modifiers, compatibilizers, and coupling agents is required to improve the intermolecular interactions between the matrix and reinforcing fibers.^63^ In a previous study,^64^ KH550 was used to modify the surface of BF before its introduction into the matrix of flame-retardant EVA/MH composites. It was observed that the mechanical characteristics were markedly improved, while retaining the flame-retardant features of the EVA/MH composites. The carbon layer produced by the EVA/MH composites is fragile and prone to fractures, creating fissures, which facilitate both heat conduction and oxygen entry. In the case of EVA/MH-BF, the synergistic influence of BF on the carbon layer allows the BF-enhanced carbon layer to shield the EVA matrix in the presence of flames, thus decelerating the transfer of heat and oxygen and diminishing the intensity of combustion (Fig. 4a). A BF/nickel alginate–brucite-based flame retardant provides a synergistic effect primarily by enhancing the charring process in the condensed phase during ignition.^65^ The fire-resistant and rigid BF incorporated into the char layer boosts the integrity of the protective barrier, which extends the duration in which the carbon-containing groups in the matrix degraded, leading to decreased heat and smoke release (Fig. 4b).

A previous study^66^ examined both the combined and individual effects of long flax fiber (LFF) bundles, short BF, and rice husk powder (RHP) on the properties of PP hybrid composites. LFF primarily provides mechanical strength, BF enhances mechanical, thermal, and flame-retardant properties by filling gaps, and RHP reinforces and improves the overall composite. Specifically, the fire performance index improved by 237.5% and the fire growth index by 101.33% compared to pure PP, highlighting significant advancements in fire safety. These enhancements are attributed to the lignin in BF and the silica in RHP.^67^ BF also had excellent heat resistance.^68^ When BF was added to PP plastic, the thermal stability of PP would be improved, and the initial decomposition temperature of PP would increase. In other words, the thermal decomposition of PP would be suppressed, thereby improving the flame retardancy of PP. BF had a very high LOI value, which was also conducive to improving the flame retardancy of PP plastics. The incorporation of BFs facilitated the formation of a char layer during the burning of PP. This expanded and porous char layer could sustain the melted PP as it burned on the established char layer, thus further enhancing the accumulation of the char layer. The char layer acted as a shield between the PP and the flame, severing the interaction between PP and the fire, while also impeding the emission of combustible gases produced by the melting of PP. This was due to the fact that BFs are made up of various metallic and non-metallic oxides. Research has indicated that TiO_2_ not only fosters the creation of char layers but also boosts the flame resistance of PP. Additionally, Fe_2_O_3_, other iron oxides, and SiO_2_ exhibited the capability to advance the flame-retardant properties of polymer materials. Therefore, the diverse oxides found in BF could enhance the flame resistance of PP plastics to some degree.

Findings demonstrated that basalt powder can be effectively used as an environment-friendly modifier for EP.^69^ The thermo-mechanical attributes of the composites were enhanced with an increase in the quantity of basalt. Composites fortified with BF and modified hydrotalcite were obtained, and the impact of the fiber and modified hydrotalcite on the microstructural features of the composites was researched.^70^ The synergistic effect is optimal when the hydrotalcite diameter resembles that of BF, resulting in a significant boost in the performance of the composite material. A combination of acetone (pre-treatment) and KH550 (formal surface treatment) for the basalt fiber (BF–AT) resulted in the EP/BF composite, which showed that the inclusion of BF–AT slightly reduced the LOI value from 26.3% to 25.1%, but still maintained its good performance during the vertical burning test, with a subsequent increase in pHRR.^71^

### 3.5. Flame-retardant polymer composites based on vermiculite

Vermiculite (VMT) is a swelling hydrous phyllosilicate clay mineral with an ideal chemical formula of (Mg^2+^, Fe^2+^, Fe^3+^)_3_[(SiAl)_4_O_10_]OH_2_·4H_2_O.^72^ These silicates consist of tetrahedral (silicon, tetracoordinate) and octahedral (aluminum, hexacoordinated) sheets that create a lamellar crystalline structure derived from a 2 : 1 mineral clay unit cell. When heated to temperatures between 650–950 °C, vermiculite can expand to as much as 30-times its initial volume, making expanded vermiculite (EV) a popular additive in materials and geopolymers for enhanced porosity and fire resistance. The flame retardancy mechanism of VMT has been studied and documented by numerous researchers.^73^ One of the most important reasons for the fire resistance of VMT is its composition, as given in Table 1. SiO_2_, Al_2_O_3_, MgO, and Fe_2_O_3_ are the main components of VMT. It has been reported that SiO_2_, Al_2_O_3_, MgO and Fe_2_O_3_ have high thermal stability, and therefore contribute to improving the flame retardancy and reducing the smoke emission of polymer composites. The flame retardancy mechanism was not only attributed to the VMT composition but also residual foam formation during burning. The formation of a multilayered, foamy, carbon char layer with good heat and oxygen impermeability resulted in reduced flame spread during the VMT test.

The formation of polyurethane/vermiculite foam composites (PU/VMT) was controlled based on the percentage of clay added in the formulation.^72^ Upon the integration of VMT, the surface area of the foams decreased, obstructing the airflow of oxygen, and as a result the combustion process. Therefore, it is deduced that at that point, the PU mass ratio per unit volume decreased with an increase in the amount of VMT. Consequently, it requires additional oxygen to sustain the combustion following the addition of clay. It should also be noted that excess VMT prevents homogenization in the matrix, which leads to the formation of an uneven residual carbon layer after firing, accompanied by a decrease in firing with an increase in the VMT content above 15%. Polyurethane-imide (PUI) foams were obtained using the polyimide prepolymer method, in which polyurethane endowed the compound with mechanical properties and polyimide imparted flame retardancy.^74^ EV, which has a broader layer spacing compared to VMT, served as a filler to further improve the mechanical and thermal characteristics of the PUI foams. The addition of EV resulted in a substantial increase in the thermal stability of the polymer composite. The LOI increased from 24% to 30.8% as the amount of EV increased from 0% to 9.6%. Consequently, the application scope of PUI foams could be greatly broadened with the incorporation of EV. In a previous study,^75^ the authors assessed, through CC tests, the performance of magnesium urea complexes and VMT (urea–VMT) as independent flame retardants in polyurethane (PU) resin and flexible PVC. The urea–VMT created an exfoliated protective barrier layer, which facilitated the thermal stabilization of the condensed phase. The emission of halogen compounds from PVC, coupled with the effect of the exfoliated barrier layer, inhibited the creation of a combustible air-fuel blend. Alternatively, in the PU composites, urea–VMT was unable to form a cohesive protective barrier layer.

To enhance the distribution of organically modified VMT (OVMT) within polymethylmethacrylate (PMMA), the technique of ultrasonic *in situ* polymerization was utilized.^76^ The obtained nanocomposites showed better mechanical and thermal properties than those prepared without the assistance of ultrasonic irradiation. Nanocomposite materials were produced from the blend (50/50) of polybutadiene rubber and ethylene propylene diene monomer rubber (EPDM) as the matrix and OVMT, respectively. The rubber nanocomposite sheets were irradiated (25, 50, 75, 100 and 150 kGy) using the γ-radiation technique as a crosslinking tool.^77^ The influence of two natural minerals, perlite and VMT, on the properties of ethylene–propylene rubber composites was investigated.^78^ According to the CC data, it is clear that the flammability parameters were significantly reduced by the incorporation of the studied minerals into the polymer matrix.

**Fig. 5:**
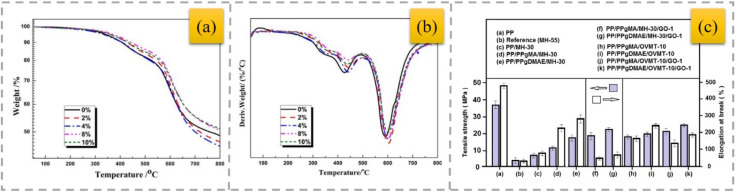
TG (a) and DTG (b) curves of polyamide foams with different VMT contents,^79^ reproduced from ref. 79 with permission from John Wiley and Sons, Copyright 2017. Mechanical properties of neat PP, reference sample and composite materials (c),^81^ reproduced from ref. 81 with permission from John Wiley and Sons, Copyright 2020.

Flexible polyimide composite foams were obtained using dianhydride and isocyanate as the initial substances by first pre-dispersing VMT in isocyanate.^79^ With the addition of VMT, the thermal stability and flame resistance of the polyimide foams improved (Fig. 5a and b, respectively). VMT possesses excellent heat insulation and high temperature stability. During high-temperature processing, the filler blocks heat transfer in the bulk foam. Therefore, filling with VMT can apparently further enhance the mechanical and thermal properties of polyimide foams.

Polyamide 11-based bionanocomposites were prepared by melt compounding with 10 wt% clays of different chemistries and morphologies.^80^ The performance of the VMT clays in terms of mechanical reinforcement and flame resistance was assessed against organo-modified montmorillonite and needle-shaped sepiolite. In comparison to the unmodified polymer, all the clay types decreased the pHRR and the smoke emission rate during CC. Surprisingly, the needle-like sepiolite and the two VMT types surpassed the montmorillonite organoclay in fire performance, despite the latter exhibiting the highest level of exfoliation within the polymer matrix.

The influence of graphene oxide (GO) modified with 3-amino-propyl-triethoxy-silane (APTS), OVMT, and MH mixtures on the flame-resistance characteristics of PP was investigated.^81^ PP grafted with MA (PP–gMA) and PP grafted with amine–alcohol(PP–gDMAE) were used as compatibilizers to improve the compatibility of the polymer matrix with the filler. PP–gDMAE enhanced the exfoliation of OVMT with a minor increase in the interlayer spacing; however, mixtures of OVMT and modified GO did not considerably boost the flame-resistant and mechanical characteristics in comparison to the impact of MH and modified GO mixtures (Fig. 5c). Research^82^ illustrates that a VMT–polymer composite film, although it is quite thin, can effectively hinder the transfer of heat without breaking down for more than just a fleeting moment when exposed to direct flames. VMT is a thermally insulating agent that can withstand flames up to about 1200 °C. Direct fire testing revealed that with a VMT concentration of around 75 wt%, films measuring 20 µm in thickness can endure flames for over a minute. In the absence of VMT, the polymer film is incinerated instantly upon flame contact.

### 3.6. Flame-retardant polymer composites based on montmorillonite

Montmorillonite (MMT), a natural layered mineral, has been widely used in many fields because of its advantages of low cost, cation-exchange capability, and naturally abundance.^83^ MMT has been widely investigated as a mineral filler for IFRs due to its ability to reduce the flammability of polymers, even when added in small amounts to polymer composites. When considering the benefits of MMT, it is crucial to emphasize not just its capacity to decelerate thermal conduction between the environment and the polymer substance, but also its effectiveness in minimizing the emission of volatile compounds into the gaseous phase.

**Fig. 6:**
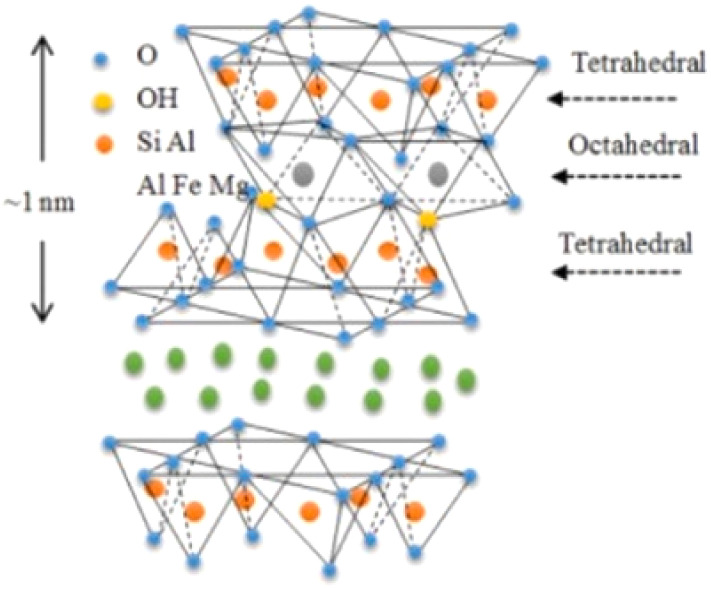
Schematic view of the structure of montmorillonite,^85^ reproduced from ref. 85 with permission from Elsevier, Copyright 2023.

MMT consists of structural units formed by two silica tetrahedral (T) layers with an alumina octahedral (O) layer placed in between^84^ (Fig. 6 and Table 1). These tetrahedral and octahedral layers are organized so that the apexes of the tetrahedrons from each silica layer and one of the hydroxyl strata from the octahedral layer unite to create a shared layer.

The atoms within this layer, which are affiliated with both layers, are solely oxygen instead of hydroxyl. Consequently, it is classified as a three-layer clay mineral comprised of two silica tetrahedral layers enclosing a central alumina octahedral layer, with T–O–T layers forming the fundamental structural unit.

**Fig. 7:**
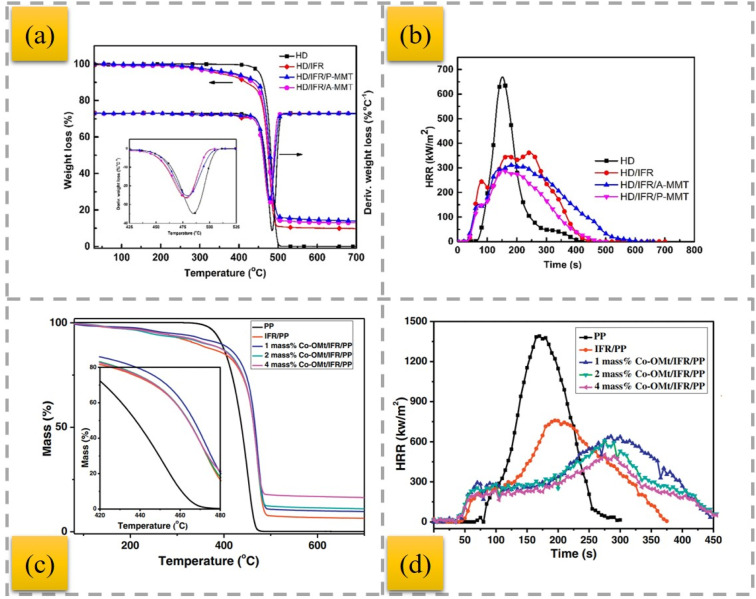
TGA and DTG curves (nitrogen) (a) and HRR (b) of the IFR–HDPE composites,^86^ reproduced from ref. 86 with permission from John Wiley and Sons, Copyright 2022. TGA (nitrogen) (c) and HRR (d) curves of pure PP and various PP composites,^92^ reproduced from ref. 92 with permission from Elsevier, Copyright 2017.

MMT was modified with dodecyl trimethyl ammonium bromide (DTAB) and tetraphenyl phosphonium bromide (TPB), and then incorporated with IFR into HDPE composites.^86^ The char residue of the flame-retardant composites contained the modified MMT (Fig. 7a). MMT can improve the thermal stability of polymer/clay nanocomposites due to its excellent barrier properties. The use of modified MMT contributes to the formation of compact char residues with a graphitic structure, which can act as a barrier to heat, oxygen and other combustible volatiles, and thus enhances the flame-retardant properties (Fig. 7b).

Nano MMT modified with silane and reinforced HDPE/wood fiber composites were prepared.^87^ Unlike the mechanical properties of the composite, which initially increased, and then decreased with an increasing amount of added nano-OMMT (organically modified montmorillonite), the flame-retardant properties and thermal stability significantly increased with increasing nano-OMMT amounts. Composites based on recycled high-density polyethylene (RHDPE), marula seed cake (MSC) and OMMT were prepared.^88^ The effects of MSC and OMMT on their combustion were studied. OMMT increased LOI and TTI but decreased HRR, mass loss rate and burning rate (BR). The combination of OMMT, MH, GO and EG as IFR for linear low-density polyethylene-cyclo-olefin copolymer (LLDPE/COC) blends was investigated.^89^ The compatibilizer was discovered to enhance the distribution of fillers and elevate the LOI to 22% for clay, 23% for GO, and 26.5% for EG composites. The findings of this study indicated that utilizing each additive together allows the overall MH filler proportion to be decreased from 55% to 20% to meet the flame-retardant standards. A single-molecule IFR, melamine salt of montmorillonite phosphate (MMP), was synthesized.^90^ A study on the synergistic effect of MMP and ZnB on the thermal stability and flammability characteristics of LLDPE showed that the addition of MMP and MMP/ZnB to LLDPE increased the thermal stability of the composite at high temperatures and increased char formation at 750 °C. The composite of LLDPE containing 30% MMP/5% ZnB had a V-0 rating in the UL-94V test. Also, MMP and MMP/ZnB additives reduced the fire risks of LLDPE, which was confirmed by the fire performance index, fire growth rate index, and maximum average rate of heat emission data. The synergistic effect of the melamine salt of chitosan phosphate (MCHP) and OMMT on the thermal stability and flammability properties of LLDPE was studied.^91^

The TGA of a range of LLDPE composites indicated that incorporating OMMT into MCHP significantly enhanced the thermal stability of LLDPE, while also increasing the char residue. The results of the flame retardancy evaluation of LLDPE showed that the inclusion of 1 wt% OMMT in 30 wt% MCHP contributes to achieving a V-0 rating in the UL-94 test. Moreover, this resulted in a significant reduction in pHRR, THR, CO and CO_2_ emissions, and fire growth index (FGI). *i.e.*, a reduction in the fire hazard of LLDPE.

To enhance the flame resistance of IFR/PP composites, OMMT intercalation cobalt compounds (Co-OMt) were synthesized and treated with acidified chitosan to further increase the interlayer distance in MMT.^92^ The incorporation of 4 wt% Co-OMt led to the formation of 4 wt% Co-OMt/IFR/PP nanocomposites, achieving a UL-94 V-0, with a remarkable LOI value reaching 32.1%. The addition of Co-OMt remarkably increased the amount of residual layers at high temperature, indicating that Co-OMt had an excellent catalytic charring performance (Fig. 7c). In contrast to the IFR/PP composites, Co-OMt/IFR/PP nanocomposites containing 1 wt%, 2 wt%, and 4 wt% Co-OMt exhibited a slower combustion rate, and the pHRR further declined to 640, 609 and 503 kw m^−2^, respectively (Fig. 7d).

**Fig. 8:**
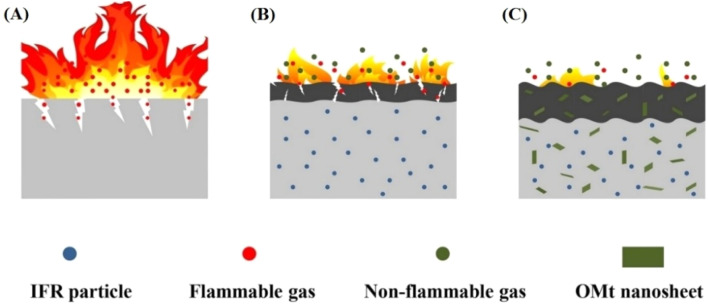
Schematic of flame-retardant mechanism of (A) pure PP, (B) PP/IFR_APP_ and (C) PP/IFR_APP/OMt_,^100^ reproduced from ref. 100 with permission from John Wiley and Sons, Copyright 2022.

OMMT was utilized as a synergist for an IFR that was constructed from APP and a hyperbranched charring foaming agent (HCFA) to enhance the retardant efficiency of PP.^93^ The findings revealed that incorporating 20% IFR with OMMT had a beneficial impact and enhanced the flame resistance of the PP composites. Notably, the inclusion of 2 wt% OMMT significantly enhanced the LOI parameters of the PP systems loaded with 20% total flame retardants from 29% to 31.5%, allowing the samples to achieve a V-0 rating, along with a decrease in HRR, THR, CO_2_, and CO emissions. Nano-Sb_2_O_3_ particles (nano-Sb_2_O_3_), MMT, and brominated polystyrene (BPS) were utilized to increase the flame resistance of PP, where nano-Sb_2_O_3_ underwent modification with cetyltrimethylammonium bromide and polyethylene glycol, while MMT was treated with a silane coupling agent.^94^ The findings indicate that MMT can considerably enhance the thermal stability and flame resistance of the PP matrix by strengthening the char layer and creating a barrier effect. In comparison to the 3.5% nano-Sb_2_O_3_/8% BPS-PP composites, the LOI of the 3.5% nano-Sb_2_O_3_/3% MMT/8% BPS-PP composites improved from 26.9% to 29.0%, achieving a UL94 rating of V-0. To improve the flame-retardant properties of PP, MMT/nano-Sb_2_O_3_/BEO/PP-based composites were prepared. The incorporation of 13 wt% of BEO, 7 wt% of nano-Sb_2_O_3_ and 7 wt% of MMT within the PP matrix resulted in excellent frame retardant properties, where the LOI value of the MMT/nano-Sb_2_O_3_/BEO/PP composites increased to 27.9% and their flame-retardant grade reached V-0 in UL-94 testing.^95^ APP was combined with aluminium diethylphosphonate (ADP) in a mass ratio of 4 : 1, blended with dipentaerythritol (DPER) to create an IFR, and OMMT was incorporated as a synergistic flame retardant, followed by melt blending to produce IFR PP composites.^96^ The results indicated that incorporating OMMT into IFR at the appropriate levels can yield a beneficial synergistic effect on flame retardancy. APP and MMT are widely used as flame-retardant additives for PP, but their low synergistic flame-retardant efficiency and poor compatibility with the PP matrix require further research in this direction. In a previous study,^97^ APP and MMT were encapsulated in the one microcapsule (PU@A-M) at the determined optimal ratio through the “bridging” reactions of diethylenetriamine (DETA) with APP and MMT. Compared with the PP/A + M composites with a physical mixture of APP and MMT, the LOI, pHRR, THR, and TSP of PP/PU@A-M decreased by 5.7%, 48.8%, 3.1%, and 20%, respectively. A linear polymeric charring agent (PEPAPC) was created through a nucleophilic substitution reaction and later integrated into the PP substrate to enhance its flame resistance and anti-dripping capabilities.^98^ Unfortunately, the opposing polarity between the IFR and the polymer matrix could severely damage the interfacial compatibility, adversely affecting the flame-retardant effectiveness and smoke toxicity reduction in the PP/IFR composites. When 2 wt% well-dispersed OMMT was incorporated, it resulted in a significant reduction in pHRR and THR (90.5% and 62.7%) compared with pristine PP, and the increase in LOI from 18.5% for the original PP to 32% can be ascribed to the nano-barrier and catalytic carbonization effect of evenly distributed OMMT within the polymer matrix. More notably, the well-distributed OMMT demonstrates a remarkable reduction in smoke toxicity and toughening and strengthening effect on the PP/IFR composite. An MINC–MMT intercalation nickel compound was synthesized. To produce organic MINC (OMINC), cetyltrimethylammonium bromide was utilized for modification.^99^ OMINC was further incorporated into the PP/IFR system to prepare PP/IFR/OMINC nanocomposites. The UL-94 and LOI findings indicate that the PP/IFR/OMINC nanocomposites exhibit outstanding flame resistance, achieving an LOI value of 29.5% and UL-94 V0 rating for the PP/IFR/4 wt% OMINC nanocomposites. The addition of OMINC resulted in an efficient reduction in the flammability parameters, such as pHRR, THR, and SPR. APP-intercalated organic montmorillonite (APP/OMt) was synthesized.^100^ Moreover, an IFR composed of APP/OMt, pentaerythritol (PER) and melamine was added to PP to fabricate PP/IFRAPP/OMt composites. When the content of flame retardants added was 30%, the LOI value increased to 33.3%, and the UL-94 assessment achieved a V-0 level with just 20% added. Notably, in contrast to its IFRAPP counterpart, incorporating IFRAPP/OMt significantly reduced the smoke emitted by PP during combustion, and the quality of the carbon layer post-combustion was markedly enhanced (Fig. 8). The improved flame resistance and mechanical strength were due to the synergistic interaction between the OMt and IFR particles, along with the better dispersion of IFRAPP/OMt within the PP matrix.

In a previous study,^101^ a comprehensive investigation was carried out on the combined effects of a phenethyl-bridged DOPO derivative (PN–DOPO) and OMMT on the flame retardancy and thermal and mechanical characteristics of PP composites. The findings indicated that the incorporation of 20 wt% PN–DOPO alongside OMMT enhanced the fire resistance of the PP composites. Notably, the addition of 17 wt% PN–DOPO and 3 wt% OMMT increased the LOI value of the PP matrix from 17.2% to 23.6%, allowing the sample to achieve a V-0 rating, while decreasing both the heat release rate and THR. Alongside the analysis of the char layers, the incorporation of OMMT facilitated the development of more uniform and dense structural layers featuring an aluminum–silicon barrier and phosphorus-infused carbonaceous char within the condensed phase. Consequently, OMMT enhances the flame retardancy and thermal and mechanical characteristics of PP. Additionally, combinations of PN–DOPO and OMMT demonstrated effectiveness as a synergistic system for enhancing the flame retardancy of PP composites. Research has explored a method utilizing PP/OMMT/poly(ethylene-*co*-octene) (POE), along with POE modified with maleic anhydride (POE-g-MAH) and POE grafted with both MAH and 2-hydroxyethyl acrylate (POE-g-MAH/HEA) nanocomposites.^102^ The compatibilizer substantially enhances both the impact strength and thermal stability of the PP/OMMT nanocomposites, while also successfully preventing a decline in their tensile strength.

The combined influence of halloysite nanotubes (HNTs) and MMT nanoclay nanoparticles was investigated for their impact on flame retardancy and mechanical strength in an intumescent APP-based PP/kenaf composite.^103^ TGA revealed that incorporating HNTs led to a higher decomposition temperature. Conversely, the addition of MMT generally resulted in a reduced maximum decomposition temperature when tested under an inert environment. Flammability testing within the IFR system revealed that nanoparticles, due to their ability to exfoliate, significantly decrease sustained burning. Researchers modified the MMT intercalation iron compound (MIIC) by incorporating cetyltrimethylammonium bromide, resulting in the creation of organic MIIC (OMIIC).^104^ PP/aluminum hydroxide (ATH)/OMIIC nanocomposites were obtained using a melting intercalation method. The combustion evaluations indicate that the addition of a minor amount of OMIIC in the PP/ATH systems can significantly enhance their UL94 performance and reduce dripping occurrences. HRR is notably lowered due to the creation of nanocomposites, and the THR of the PP/ATH/OMIIC nanocomposites was found to be lower compared to the PP/ATH nanocomposites, which can be attributed to the development of a dense protective char layer. An IFR PP system made up of a charring agent (SBCPO), APP, and PP matrix was examined for the synergistic effects of OMMT on flame retardancy and thermal degradation.^105^ According to the experimental results, the composites could pass the UL-94 V-0 rating and the LOI value of the PP/IFR system could be greatly increased to 29.5% with a tiny amount of OMMT. The synergistic effect of OMMT and IFRs on encouraging the formation of a more compact and continuous char layer, which improved the barrier action to heat, oxygen, and flammable gases, was the primary cause of the flame-retardant mechanism, according to the char structure study. Additionally, the TGA data showed that OMMT could effectively improve the thermal stability of the PP/IFR composites. To examine the impact of acidic site strength and nature (Brønsted and Lewis) on the synergistic action with an intumescent formulation made of APP and pentaerythritol (PER) when incorporated into a PP matrix, raw MMT was exposed to varying acidic activation times.^83^ In the LOI test, the flammability attributes reached a maximum of 38% for the system with more moderate-strength Brønsted sites than the Lewis ones. This finding implies that these Brønsted acidic sites are crucial because they participate in the esterification process between APP and PER, which results in the creation of char.

The layer-by-layer technique was applied to form multilayered protective coatings for polyamide 6/MMT (PA6/MMT) hybrid nanocomposites.^106^ It was found that this method could be successfully used to improve the flammability characteristics of PA6-based composites. The surfaces on which the development of interwoven fibrous crystal structures was seen had a propensity to shield the entire material from the damaging effects of heat, helping to lower the maximum point of HRR, among other things, according to microscopic investigations. The effects of an MH/MMT hybrid on the flame retardancy, thermal, and thermo–mechanical properties of PA6/PP nanocomposites were investigated.^107^ Maleated PP was utilized as a compatibilizer, and MMT was partially substituted for MH at a total filler content of 30 wt%. Analysis of mass loss *via* measurements using a calorimeter revealed that the pHRR, total heat, and TPR values decrease in the presence of MMT. This decrease is due to the formation of a protective surface and insulating MMT layer within the MgO layer formed on the surface during combustion. The creation of a thicker, more compact residue layer that serves as an insulating barrier and lowers the yield of volatile breakdown products is the mechanism of flame protection.

To reduce the flammability of flexible polyurethane (FPU) foam, chitosan–montmorillonite nanosheet (CH–MMTNS) membranes with varying MMTNS thicknesses were created.^108^ It was discovered that a thinner MMTNS membrane reduced the flammability of the FPU foam more effectively. This was due to the ability of the MMTNS membrane to deposit cheek by jowl and create a dense CH–MMTNS barrier on the foam surface, which significantly reduced the transfer of volatile gases, heat, and oxygen. The effect of an IFR and MMT on the fire resistance of TPU was investigated.^109^ The LOI of TPU composites increased from 17.2% to 28% with 22% IFR and 3% OMMT fillers, leading to a UL-94 V-0 grading. The combination of 22% IFR and 3% OMMT produced the least amount of smoke and heat.

**Fig. 9:**
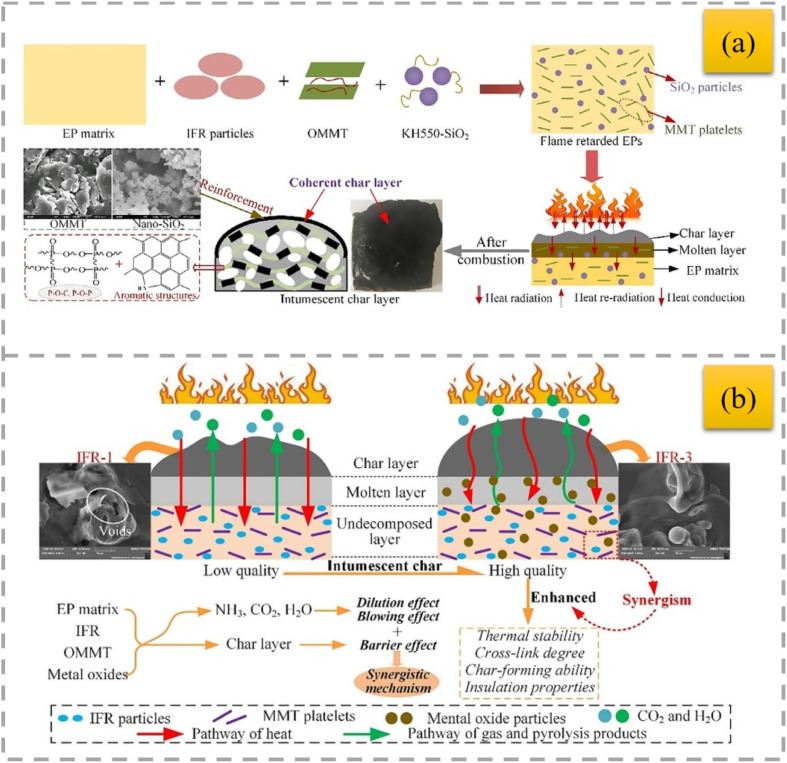
Possible synergistic flame-retardant mechanism of OMMT/nano-SiO_2_ for the intumescent flame-retarding EPs (a),^110^ reproduced from ref. 110 with permission from John Wiley and Sons, Copyright 2020. Synergistic mechanism of IFR-OMMT- metal oxide ternary system in EPs (b),^111^ reproduced from ref. 111 with permission from John Wiley and Sons, Copyright 2021.

The impact of OMMT and nano-SiO_2_ together on the fire behavior of intumescent flame-retarding EPs was assessed.^110^ Fire test results demonstrate that the combination of IFR, OMMT, and nano-SiO_2_ has a remarkable synergistic effect on enhancing the flame retardancy and smoke suppression qualities of EPs. When OMMT and nano-SiO_2_ are combined, they have a greater synergistic effect on the char-forming and fire safety of the intumescent flame-retarding EPs than when they are used separately. This phenomenon can be explained by the uniform dispersion of OMMT and nano-SiO_2_ particles, which can help build a more compact and continuous intumescent char that effectively reduces fire dangers by preventing the passage of heat and combustible gases (Fig. 9a). The synergistic effects of OMMT with various metal oxides (Bi_2_O_3_, Sb_2_O_3_, and MoO_3_) on the improved fire safety of intumescent flame-retarded EPs were assessed.^111^ In comparison to IFR or IFR-OMMT systems, tests reveal that the IFR-OMMT-metal oxide ternary system can provide EPs with higher synergistic efficiencies on the enhancement of flame retardancy, smoke suppression properties, and charring ability (Fig. 9b). The synergistic efficiency follows the order of IFR/OMMT/Sb_2_O_3_ > IFR/OMMT/MoO_3_ > IFR/OMMT/Bi_2_O_3_.

FeCu-montmorillonite (FeCu-MMT) modified with hexadecyltrimethylammonium bromide (CTAB) and Fe polycations to prepare multiple modified FeCu-MMT (mm-FeCu-MMT) was synthesized to increase the fire safety of EP at a lower loading.^112^ The EP matrix was combined with mm-FeCu-MMT and 9,10-dihydro-9-oxa-10-phosphaphenanthrene-10-oxide (DOPO) to create EP mm^−1^-FeCu-MMT and EP mm^−1^-FeCu-MMT/DOPO nanocomposites. Because of its good physical barrier effect, low cost, and abundant availability, it can be employed as a flame retardant. EP/1 wt% mm-FeCu-MMT/3 wt% DOPO nanocomposites reached the V-0 level, while EP/4 the wt% DOPO composites pass the UL-94 V-1 level. As flame retardants, DOPO-MMT, DOPO, MMT, and physical combinations of DOPO + MMT were used to obtain EP composites.^113^ Among the EPs with DOPO, MMT, and physical mixture of DOPO + MMT, the EP/DOPO-MMT nanocomposites demonstrated the best flame retardancy, with the flame-retardant grade of V-0. DOPO-MMT was uniformly distributed throughout the EP as nanosheets in the EP/DOPO-MMT composite. Compared to physically combined DOPO + MMT, DOPO, and MMT, DOPO-MMT contributed the most to the flame retardancy in the EP composites in the flame retardancy test. The V-0 grade was also attained by the EP/DOPO-MMT composite. Because of the improved dispersity of MMT layers in the EP matrix, the DOPO-MMT nanocompound exhibits greater flame retardancy, as demonstrated by the lighter color and improved transparency of the EP samples and the smoother and less porous structure of their chars.^114^ Melamine hydroxy ethylidene diphosphonate-wrapped montmorillonite was effectively manufactured through *in situ* fabrication in a previous study^115^ to provide EP with reduced fire dangers. Significantly less harmful CO and organic volatiles were released throughout the breakdown process, suggesting reduced toxicity and increased fire safety.

By melting intercalation, a Schiff-base polyphosphate ester (PAB)-functionalized MMT (PAB–MMT) IFR was integrated with PAB to incorporate in EVA.^116^ The findings demonstrated that the flame retardancy of the EVA/PAB composite increased when 5.0 wt% PAB–MMT was substituted for the equivalent amount of Na–MMT. Compared to pure EVA or composites including PAB or Na-MMT/PAB, this composite had a higher LOI value and a shorter igniting time in the UL-94 rating. OMMT and graphene nanosheets (GNSs) were used to create a halogen-free intumescent flame-retardant EVA/IFR system with improved thermal-oxidative resistance at high temperatures and a well-dispersed structure.^117^ Remarkably, the EVA/IFR composite including both OMMT and GNSs showed the strongest flame retardancy with the lowest pHRR value of 529.58 kW m^−2^ and the highest LOI value of 24.8%, while the amount of residual chars increases to 12.7% at 700 °C according to TGA. The development of a comprehensive and compact protective char layer is responsible for the exceptional flame retardancy. To develop flame-retardant and relatively green cable coating materials, PE was melt-blended with natural calcium MMT (C–Ca) pre-dispersed in EBA (ethylene–butyl acrylate copolymer), EVA, or mEVA (EVA modified with maleic anhydride).^118^ Organophilized montmorillonite (CW9) was examined for comparison. Both clays were not completely exfoliated in the matrix, according to the primary investigation of composites with EBA/C–Ca, EVA/C–Ca, and mEVA/CW9 pre-dispersions; nonetheless, C–Ca (7.5 wt%) significantly increased the LOI from 18% O_2_ (PE) to 22.0% O_2_. The sample containing 10% of CW9 had a slightly higher LOI value (22.2% O_2_).

The impact of two flame retardants [APP and MMT] on the mechanical features, physical properties, and flame retardancy of polycarbonate (PC)/ABS blends was investigated.^119^ According to this study, the intumescence effect of the flame retardants used led to a considerable increase in the LOI of the resulting PC/ABS blends due to the increased APP and MMT loadings. Although the inclusion of MMT caused the PC/ABS polymer matrix to intercalate into the interlayer galleries of MMT particles, the addition of APP enhanced the LOI through the intumescence effect. Blends of MMT and ABS were made.^120^ The flame spread rate of ABS significantly decreased with the addition of MMT, particularly when burning without a sidewall. By increasing the CO production in well-ventilated environments, MMT makes the effluent more hazardous. The impact of MMT content on the flame retardancy and physico–mechanical characteristics of PC/ABS blends exposed to electron beam radiation was studied.^121^ Due to the induction of char residues created by the presence of MMT particles, as determined by TGA analysis, it was found that an increase in MMT loading level gradually increased the flame retardancy and thermal stability of the flame-retarding PC/ABS blends. Additionally, by creating crosslinking networks in PC/ABS blends that limit the permeability of air and volatiles *via* the polymer matrix, electron beam irradiation progressively increased the flame retardancy.

**Fig. 10:**
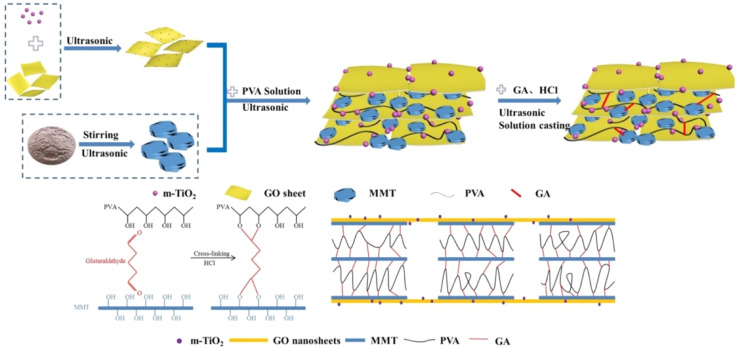
Schematic of the film preparation process of the *m*-TiO_2_@GO/MMT/PVA nanocomposite,^125^ reproduced from ref. 125 with permission from John Wiley and Sons, Copyright 2021.

Glass-fiber-reinforced polyamide 6 T (GFPA6T) was made more flame-resistant by combining OMMT with a DOPO derivative (PN-DOPO).^122^ The findings show that 2 wt% OMMT and 13 wt% PN-DOPO in GFPA6T received UL-94 V-0. When OMMT was added, the pHRR and TSR significantly dropped in comparison to GFPA6T/15 wt% PN-DOPO. According to the TGA data, the residual mass and thermal stability of the samples both significantly increased as the amount of OMMT increased. The flame retardancy and smoke suppression of GFPA6T were enhanced by the combination of PN-DOPO and OMMT without sacrificing its mechanical qualities. OMMT and one-dimensional needle-like ZnO were used as flame retardants in polystyrene (PS).^123^ The flame retardancy and dynamic mechanical characteristics of the polystyrene/OMMT/ZnO nanocomposites improved as a result of the synergistic impact between OMMT and ZnO. It has been demonstrated that the PS/MH/OMMT composite, when degraded in air or burned in a flame, can create a more continuous and compact layer of charred residue than the PS/MH composite.^124^ The combination of MH and OMMT makes the composite more difficult to ignite and decreases the release of toxic gas. A straightforward solution casting technique was used to create modified TiO_2_ (*m*-TiO_2_)@graphene oxide (GO)/MMT filler-reinforced polyvinyl alcohol (PVA)-based nanocomposite films (Fig. 10) with superior gas barrier, flame retardant, and mechanical properties.^125^ The flame-retardant properties of the *m*-TiO_2_@GO/MMT/PVA nanocomposite films are excellent, where they self-extinguish after the flame propagation has ceased. However, when a pure PVA film is used, it burns completely. Nanocomposites made from environmentally friendly raw materials with excellent physical and mechanical properties will find wide application in the packaging industry.

**Fig. 11:**
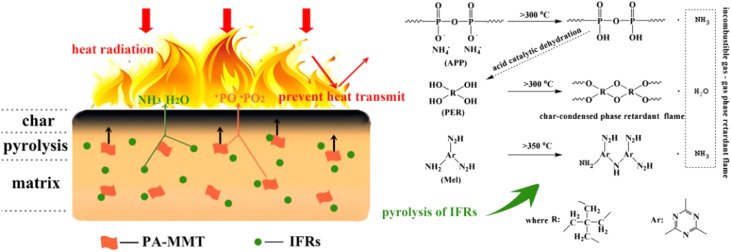
Schematic illustration of flaming UPR/IFRs/ montmorillonite‐containing phytic acid (PA‐MMT) composite,^128^ reproduced from ref. 128 with permission from John Wiley and Sons, copyright 2019.

PVC was mixed with iron oxide-modified MMT as a flame retardant, and its smoke-suppressive and flame-retardant qualities were studied.^126^ A more compact char residue developed on the surface of the sample containing iron oxide-modified MMT during the combustion process, suggesting that the iron oxide-modified MMT could lower the HRR in flame-retardant PVC. The sample containing modified iron oxide MMT exhibited greater thermal stability than pure PVC, according to the TG result. A stable bisphenol A diphenyl diphosphate (BDP) emulsion was created in water/acetone mixed solutions using a long alkyl chain quaternary phosphonium cationic surfactant.^127^ Quaternary phosphonium on the surface of the emulsion and montmorillonite exchange cations to create BDP emulsion-based montmorillonite (BMMT). BMMT and poly(styrene–ethylene–butylene–styrene) (SEBS) thermoplastic elastomers were combined to create nanocomposites. BDP and nanoclay flakes produced dense carbon during combustion, according to flame-retardant tests, and the nanocomposite exhibited strong hydrolysis resistance and flame retardancy. After its synthesis by acid treatment, modified montmorillonite-containing phytic acid (PA-MMT) was added to unsaturated polyester resin (UPR) with an IFR.^128^ The LOI value of the UPR/IFR/PA-MMT composites increased to 29.2%. The results indicated that the incorporation of PA-MMT and IFRs significantly improved the combustion behavior of UPR (Fig. 11).

Table 3 presents some results on the influence of montmorillonite on the flammability of polymer composites.

**Table 3:** Fire properties of montmorillonite-based polymer composites

Formulation of composites		LOI	UL-94	PHRR (kW m^−2^)	Reference
Matrix	Additive				
HDPE + 3PE-g-MAH	—	18.1 ± 0.1		670 ± 6	86
	12.5IFR(APP : THEIC = 3 : 1)	25.1 ± 0.1		362 ± 12	
	11IFR + 1.5AMMT	24.6 ± 0.3		311 ± 17	
	11IFR + 1.5PMMT	25.8 ± 0.2		288 ± 14	
LLDPE	—		NR	1318	90
	30% MMP + 5% ZnB		V-0	374	
LLDPE	—		NR	1318	91
	30% MCHP + 1% OMMT		V-0	310	
PP	—	17	NR	1390	92
	23% IFR	26.5	V-2	759	
	19% IFR + 4% Co-OMt	32.1	V-0	503	
PP	—	17.0	NR	906	93
	15% APP + 5% HCFA	29.0	V-1	143	
	13.5% APP + 4.5% HCFA+2% OMMT	31.5	V-0	55.2	
PP	—	17.4	V-2		94
	3.5% nano-Sb_2_O_3_ + 8% BPS	26.9	V-0		
	3.5% nano-Sb_2_O_3_ + 3% MMT + 8% BPS	29.0	V-0		
PP	—	17.4	V-2		95
	7% nano-Sb_2_O_3_ + 16% BEO	26.6	V-0		
	7% MMT + 7% nano-Sb_2_O_3_ + 13% BEO	27.9	V-0		
PP	—	18.5%	NR	867	98
	20% PP-g-MAH + 13.5% APP + 4.5PEPAPC + 2% OMMT	31.5	V-0	153	
	10% PP-g-MAH + 13.5% APP + 4.5PEPAPC + 2% OMMT	32	V-0	82	
PP	—	17.5 ± 0.2	NR	1457	99
	24% IFR	26.8 ± 0.2	V-1		
	20% IFR + 4% OMINC	29.5 ± 0.2	V-0		
PP	0.5% antioxidant	18.5	NR		100
	0.5% antioxidant + 30% IFR_APP/OMt_	33.3	V-0		
PP	—	17.2	NR	1260.1	101
	20% PN-DOPO	23.1	V-0	1162.3	
	17% PN-DOPO + 3% OMMT	23.6	V-0	914.0	
	15% PN-DOPO + 5% OMMT	24.0	V-1	976.4	
	7% OMMT	20.3	NR	1296.2	
PP	—	17.3	NR	1425	104
	50% ATH	23.6	NR	539	
	45% ATH + 5% OMIIC	29.0	V-0	329	
TPU	—	17.2 ± 0.2	NR	1058.71 ± 115.38	109
	25 IFR (80% AlPi/20% triazinyl macromolecule char-forming agent)	29.8 ± 0.2	V-2	346.12 ± 24.06	
	22 IFR + 3 Na-MMT	28.5 ± 0.2	V-2	291.34 ± 31.30	
	22 IFR + 3 OMMT	28 ± 0.3	V-0	231.27 ± 20.84	
EP	—	19.2	NR	1293.3	110
	30% IFR	25.7	V-2	397.0	
	27% IFR + 3% OMMT	26.0	V-2	340.1	
	27% IFR + 1.5% OMMT + 1.5% KH550–SiO_2_	28.2	V-0	382.9	
EP	—	19.2	HB	1293.3 ± 43	111
	30% IFR	25.7	V-2	397.0 ± 27	
	27% IFR + 3% OMMT	26.0	V-2	340.1 ± 30	
	27% IFR + 1.5% OMMT +1.5% Bi_2_O_3_	27.9	V-0	321.7 ± 23	
	27% IFR + 1.5% OMMT + 1.5% Sb_2_O_3_	28.5	V-0	254.6 ± 19	
	27% IFR + 1.5% OMMT + 1.5% MoO_3_	27.6	V-0	307.1 ± 17	
EP	MMT	25.0	NR	279	113
	DOPO	32.6	V-1	580	
	DOPO-MMT	32.9	V-0	654	
EP	—	25.5		962.3	115
	Mt	25.5		908.4	
	MHEDP	27.0		708.8	
	Mt@MHEDP8	28.5		657.0	
EVA	—	19.7	NR	882.2	116
	20% PAB	23.0	V-2	718.1	
	PAB + Na-MMT	23.4	V-2	706.6	
	PAB + PAB-MMT	25.0	V-2	658.9	
EVA	—	18.6	NR	2688.01	117
	22.5 g APP + 7.5 g TPU	21.4	NR	795.16	
	20.25 g APP + 6.75 TPU + 3 g OMMT	24.6	V-2	547.41	
	21.75 g APP + 7.25 TPU + 1 g GNSs	20.2	NR	613.92	
	19.5 g APP + 6.5 g TPU + 3 g OMMT + 1 g GNSs	24.8	V-2	529.58	
PS	—	17.4		861	123
	5% MMT	19.8		359	
	5% MMT + 5% ZnO	20.9		295	
UPR	—	19.1 ± 0.2	NR	605.7	128
	IFRs	27.5 ± 0.2	V-2	259.2	
	IFRs + 1.5MMT	28.3 ± 0.2	V-0	213.1	
	IFRs + 1.5PA-MMT	29.2 ± 0.2	V-0	222.9	

### 3.7. Flame-retardant polymer composites based on perlite

Perlite is a natural clay mineral, which is a form of amorphous volcanic silica glass having a considerable water content.^129^ It consists mainly of SiO_2_, Al_2_O_3_, Na_2_O, K_2_O, and water^130^ (Table 1). This material exhibits remarkable thermal stability. Upon heating between 760 °C and 1100 °C, its volume expands significantly, ranging from 7- to 16-times its original size, and it transforms into an effective thermal and acoustic insulator. Its low thermal conductivity and inherent fire resistance suggest its potential as a valuable flame-retardant additive. Perlite has proven to be an excellent flame retardant. The addition of perlite improved both the thermal stability and the combustion behavior of a polymer composite.

In addition, perlite mining has a limited impact on the environment given that no chemicals are used and no by-products are produced in perlite processing, and minimal waste is generated.^131^ These features make perlite an environmentally friendly, sustainable, versatile, and efficient natural mineral. The flame retardancy effect of expanded perlite (ExP) can be explained by two main mechanisms. The first is the condensed-phase flame retardancy mechanism due to the good char-forming ability of ExP. Secondly, the highly porous structure and high specific surface area of ExP contribute to its flame-retardant effectiveness. Porous flame retardants can absorb the released volatile combustible gases produced from the pyrolysis of polymer and reactive oxygen, limiting the availability of fuel and oxidant for combustion. Owing to its porous structure, it also restricts mass and heat transfer, which slows down the back-heat release and hinders the flaming process. Furthermore, the release of moisture trapped in the pores of ExP provides a cooling effect during combustion and dilutes the combustible gases.

R. Guliyev *et al.* investigated the flame-retardant and smoke-release behaviors of composites based on semi-rigid polyurethane foam/expanded perlite (SrPUF/ExP).^132^ The addition of 8% ExP to the SrPUF/ExP composite reduced the amount of carbon monoxide and carbon dioxide released during combustion by 43% and 37%, respectively. Studies have shown that the volume of char residue remaining after the complete combustion of the 4SrPUF composite is 2.7-times greater than the volume of char residue from the combustion of the initial SrPUF. Mineral–rubber composites based on phosphorylated butadiene rubber (PhBR), including pure ExP and modified phosphorylated expanded perlite (PhExP) as fillers, were developed.^133^ In comparison, the PhExP/PhBR composite exhibited reduced weight loss, the highest integral procedural decomposition temperature value, and a lower *T*_max_ in its DTG curve compared with the ExP/PhBR composite and the PhBR matrix. In a previous study,^134^ ExP powder was used to fabricate epoxy composites, and the effects of the filler content on the physical and thermo-mechanical properties of epoxy were investigated. The thermal conductivity showed an enhancement of 124% compared to the pure epoxy at a perlite content of 3 wt%, and the thermal stability was improved with the addition of perlite powders in the epoxy. The reduction in the burning rate was found to be insignificant with the addition of perlite powder. The synergistic effects of perlite powder in the epoxy matrix show great potential for developing high-performance composite materials.

### 3.8. Flame-retardant polymer composites based on mica

Mica belongs to a group of aluminosilicate minerals characterized by a layered structure.^135^ This substance demonstrates chemical inactivity and thermal stability up to 600 °C, at which point dehydroxylation occurs. Its properties make it suitable for use as a flame-retardant additive and char enhancer. Research^135^ explored the combined benefits of mica minerals and IFR in improving the flame retardancy of PP, aiming for a cost-effective solution. The findings revealed a synergistic flame-retardant effect when mica is incorporated into a PP/IFR blend, enhancing the performance of the IFR system. At a mica content of 6 wt%, the LOI value for the PP compound increased to 34.9%, achieving a V-0 flame rating according to UL 94 standards. However, incorporating mica into PP without an IFR system did not improve the flame-retardant characteristics of the polymer. A previous study^136^ aimed at investigating the influence of the crystalline structure of a series of clay minerals such as mica, kaolinite, and palygorskite, besides K-feldspar on the flame retardancy of ethylene-based polymer materials with and without an intumescent formulation. The intumescent formulation used was composed of APP and pentaerythritol (PER). Investigations revealed that incorporating mica, kaolinite, or palygorskite resulted in a more thermally stable intumescent layer. The crystalline framework of the clay minerals appears to influence the synergy with APP/PER. The existence of tetrahedral SiO_4_ sheets on both surfaces of the interlayer space, as found in the structure of mica, or along the internal face of the channels in palygorskite, seems to enhance the synergistic effect. The synergistic influence of flame retardants was evaluated to augment the capabilities of ceramizable composites.^137^ Composites based on SBR were used as the polymer matrix. The synergistic effect of three different types of flame retardants was investigated including melamine cyanurate, a commonly used flame retardant that promotes carbonaceous char, low softening point temperature glass frit (which has a ceramization effect), and high aspect-ratio mica (phlogopite) platelets. The findings demonstrate that the synergistic influence of ceramization-promoting fillers and melamine cyanurate was particularly prominent regarding flame-retardant characteristics, resulting in a notable enhancement in fire resistance.

### 3.9. Flame-retardant polymer composites based on talc

Overall, the inclusion of talc and a talc/IFR combination improved the heat and fire resistance of the foams. Talc, primarily consisting of SiO_2_, MgO, and CaO, along with an IFR made up of APP and pentaerythritol, was blended into the foam at diverse weight proportions.^138^ Among them, the best fire resistance was achieved with the composition including 10 wt% the IFR and 5 wt% talc. To investigate the flame-retardant characteristics of composites and to develop a novel variety of PVC materials with outstanding flame retardancy and minimal smoke production, varying amounts of antimony trioxide (Sb_2_O_3_), talc powder, hydromagnesite, and ZnB were incorporated.^139^ The findings revealed that the LOI value for all the groups exceeded 27% after the inclusion of talc powder, magnesite, and zinc borate, which substituted a portion of Sb_2_O_3_. This value was within the refractory-grade level and indicated a good flame retardancy performance. Research^140^ examined the structural effects of kaolin and talc on the mechanical, thermal-resistant, rheological, and flame-retardant features of polycarbonate (PC) composites. The introduction of just 5 phr of filler significantly improved the flame retardancy. Compared to kaolin, talc contributed to the production of polymer composites with higher mechanical properties and flame-retardant characteristics.

### 3.10. Flame-retardant polymer composites based on halloysite

**Fig. 12:**
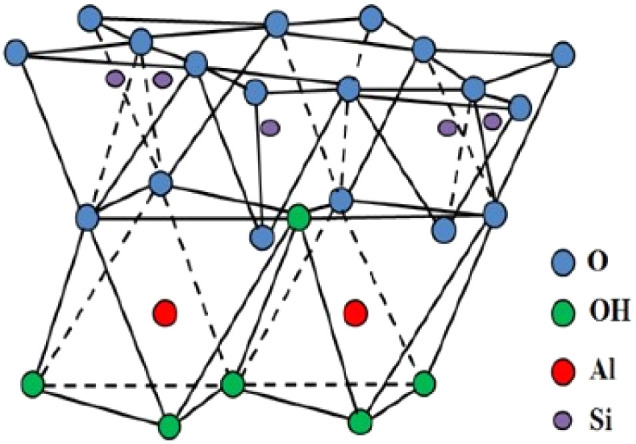
Schematic view of the structure of HNT;^145^ this article is an open access article distributed under the terms and conditions of the Creative Commons CC BY license.

Halloysite, a mineral composed of nanotubes, possesses high physical properties due to the presence of reactive sites on the inner and outer surfaces of the nanotubes.^141^ HNTs are obtained from natural deposits, and thus their use is non-hazardous to the environment. Numerous studies have examined the thermal characteristics and flame retardancy of HNTs, either independently or when incorporated as nanoparticles into a polymer matrix.^142,143^ HNTs are aluminosilicate clay minerals. HNTs, characterized by the structural formula Al_2_(OH)_4_Si_2_O_5_·*n*H_2_O, typically range in length from 0.5 to 2 µm, with an outer diameter of 30 to 190 nm and an inner diameter of 10 to 100 nm. They are composed of layers of SiO_4_ tetrahedra interspersed with layers of edge-sharing AlO_6_ octahedra^144^ (Fig. 12). The tubular configuration of HNTs arises from the contrast between the larger tetrahedral layers and the smaller Al(OH)_3_ octahedral layers.

Halloysite, a naturally occurring nanotubular material, possesses several fascinating qualities that render it an excellent choice for producing polymer-based nanocomposites suitable for engineering and functional uses.^146^ The key appealing attributes of halloysite encompass its remarkable mechanical strength/modulus, unique lumen architecture, and vital noncytotoxic properties, along with its capability to control crystallization and improve thermal stability. To form halloysite-based nanocomposites, it is introduced into polymer matrices such as thermoplastics, thermosetting polymers and elastomers. The addition of HNTs to polymer materials has various impacts on polymers. Halloysite, recognized as a conventional one-dimensional nanotube, has found extensive use in polymer composites owing to its abundant availability and affordability.^147^ HNTs, featuring a lumen-like architecture, offer both extensive internal and external surface areas for grafting modifications, along with the partial encapsulation of flame retardants within their nanotubes. To ensure the long-term aging properties of composites, HNTs are a good alternative as a carrier of flame-retardant systems and to control the migration of flame-retardant molecules.^142^ Due to the inter-tubular and interfacial interactions between HNTs and polymers as well as structure of HNTs, the thermal properties of nanocomposites are enhanced.^148^ In the last ten years, HNTs have emerged as preferred nanomaterials, functioning alongside various polymers to enhance the thermal stability and control the flame-retardant properties of the resulting nanocomposites. To increase the flame retardancy and improve compatibility between HNTs and the substrate, they could be used as a flame-retardant synergist.

The various functionalization techniques for altering nanofillers to allow interactions with polymers were discussed in a previous study.^149^ The assessment of thermal properties and fire resistance of HNTs, individually or incorporated as nanoparticles into a polymer matrix, has been explored in various studies.^141^ Nonetheless, the hydrophilic characteristics of HNTs and their incompatibility with nonpolar polymer structures have been noted as significant hurdles to achieving the adequate dispersion of platy and tubular nanoscale clays, which are the two primary geometrical forms of nanoclays. The influence of both pure and modified halloysite clays, whether used alone or alongside different types of additives, on the thermal stability and fire resistance of polymer systems has been summarized. Nanoclays have always attracted considerable attention for use in flame-retardant materials, including MMT, kaolinite, and HNTs. Environmental friendliness, low smoke generation, and non-toxicity are the key parameters governing the production of flame-retardant materials. Melamine (MEL) and bio-based phytic acid (PA) were used as the building blocks to modify HNTs using supramolecular self-assembly technology to create a novel nano-clay flame retardant (HNTs@MEL-PA).^150^ The feasibility of employing HNTs@MEL-PA as a PP flame retardant was investigated. The dispersion of HNTs@MEL-PA and its interfacial interactions with the PP matrix are significantly better than that of PP/HNT composites. HNTs@MEL-PA significantly reduces smoke production in PP and somewhat reduces its heat release. The TSP of PP was reduced with the addition of 15 wt% HNTs@MEL-PA, demonstrating its significantly increased fire safety. To create a PA/A-HNT/CS/PVA organic–inorganic composite film, amino-modified HNTs (A-HNTs), chitosan (CS), and PA were added to a polyvinyl alcohol (PVA) matrix.^151^ The weight loss rate of the composite film clearly decreased as the thermal decomposition temperature increased, and at 700 °C, the carbon residue reached 26 wt%. The LOI increased to 32.2% from 18.5%. Additionally, adding this flame-retardant system can clearly lower the combustion intensity of PVA and increase its flame-retardant grade to V-0. A reduced graphene oxide decorated with HNT (HNTs-d-rGO) hybrid composite was used as an additive to improve the flame-retardant properties of polyamide 6 (PA6).^152^ Compared to the usage of either HNTs, GO, or a combination of HNTs and GO (HNTs-m-GO) in the PA6 matrix, the incorporation of HNTs-d-rGO significantly enhanced the fire-retardant qualities of PA6. The results clearly show that the integrated HNTs-d-rGO nanostructures had higher flame-retardant activity than the simple mixture, confirming the significance of the close integration between HNTs and rGO, which is attributed to the combination of the barrier effect of rGO and the stable silica layer formed by HNTs.

### 3.11. Flame-retardant polymer composites based on kaolinite

Kaolinite is a clay mineral in the phyllosilicate family with the composition of Al_2_Si_2_O_5_(OH_4_)^54,153^ (Table 1). Kaolinite becomes colored when it contains impurities or large amounts of other minerals, such as montmorillonite and iron oxide. Kaolinite, part of the kaolin family, is classified as a 1 : 1 type of natural hydrous phyllosilicate. Its chemical structure comprises silicate layers connected to aluminum oxide/hydroxide layers, referred to as gibbsite layers. The fundamental structural unit of kaolinite consists of an octahedral layer fused with one tetrahedral sheet layer.

**Fig. 13:**
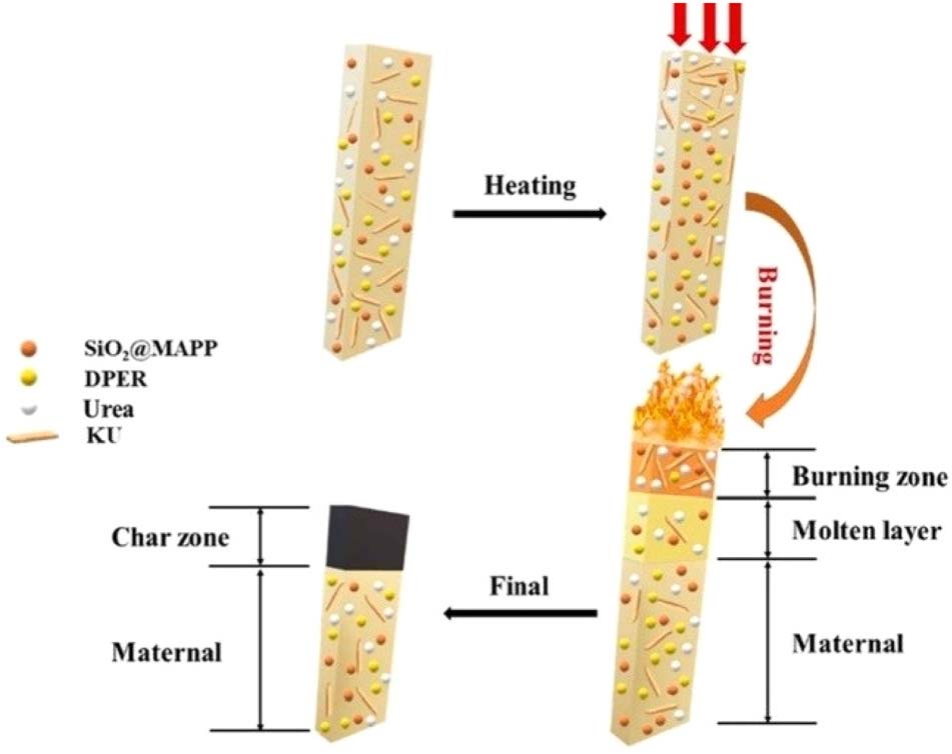
Diagram showing the combustion performance mechanism of composite materials,^155^ reproduced from ref. 155 with permission from Elsevier, Copyright 2021.

A series of articles in the literature demonstrated that IFR/Kaol is an effective synergistic flame-retardant system. However, kaolinite readily aggregates in the polymer matrix, severely impairing the mechanical performance and heat/smoke shielding function of polymer composites. To increase the compatibility between kaolinite and the polymer substrate, some surface coupling agents, such as fatty acids, titanate, and silane, have been employed.^154^ To increase the flame retardancy of low-density polyethylene (LDPE), IFR was added to modified kaolin that had been intercalated with urea (KU) *via* a mechanochemical process. As a synergistic flame retardant, KU was employed.^155^ The flame retardant properties and smoke properties of KU/SiO_2_@MAPP/DPER/LDPE composites were investigated. The LOI of SiO_2_@MAPP/DPER/LDPE increased from 24.1% to 27.2% and a UL-94 V-1 rating was achieved with the addition of 3 wt% KU. Because SiO_2_@MAPP breaks down into NH_3_ and a lot of phosphoric acid compounds during combustion, adding a certain amount of KU improved the combustion performance (Fig. 13). KU was introduced into poly(butylene succinate)/IFR by melt blending.^156^ With the introduction of KU and IFR into poly(butylene succinate), the LOI obviously increased from 21.9% to 40.1%. The pHRR decreased from 576 kW m^−2^ to 292 kW m^−2^.

**Fig. 14:**
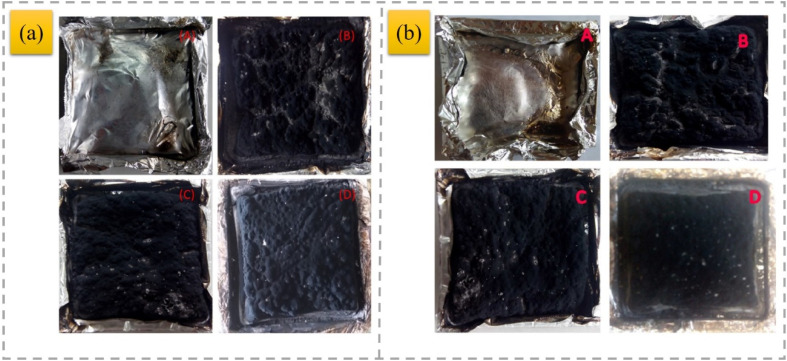
Digital photographs of the residues of (A) PP, (B) PP/IFR, (C) PP/IFR/1.5Kaol, and (D) PP/IFR/1.5Acid-Kaol (a),^157^ reproduced from ref. 157 with permission from Elsevier, Copyright 2017 and (A) PP, (B) PP/IFR, (C) PP/IFR/1.5K_0_, and (D) PP/IFR/1.5AS-K (b),^158^ reproduced from ref. 158 with permission from the American Chemical Society, Copyright 2016.

Modified kaolinite containing Lewis acidic sites (Acid-Kaol) was prepared, and then introduced into PP together with IFR. When 1.5 wt% Acid-Kaol was substituted for IFR, the LOI value of the PP/25 wt% IFR composite increased from 31.1% to 34.9%.^157^ Acid-Kaol was found to increase crosslinking during combustion and encourage the development of char, which will serve as an insulating barrier to prevent further degradation of the polymer matrix. The char became more continuous and compact after the incorporation of Kaol, especially Acid-Kaol (Fig. 14a).

Ammonium sulfamate (AS)-intercalated kaolinite (AS-K) was prepared and introduced in association with IFR into PP.^158^ It was suggested that AS-K may combine with P, N, and polyaromatic rings to create a compact structure that resembled a ceramic and would continue to burn. PP/IFR (23.5 wt%)/AS-K (1.5 wt%) attained a UL-94 V-0 rating, and its LOI value increased to 35.3%. In PP/IFR/AS-K, the char layer grew denser (Fig. 14b). In comparison to the sample containing 1.5 wt% raw kaolinite (1346 kW m^−2^), the pHRR value of the PP composite containing only 1.5 wt% intercalated kaolinite (1169 kW m^−2^) decreased by 13.2%.^159^ Thermal stability and flame retardancy were assessed, and the impact of the surface modification of kaolinite with trisilanolisooctyl polyhedral oligosilsesquioxane (POSS) in the PP composites was compared with talc.^160^ According to TGA, the addition of 30% kaolinite improved the thermal stability. CC results demonstrated that kaolinite can greatly reduce the pHRR but not unmodified kaolinite. To enhance the fire performance, raw kaolinite (Kaol) was converted into a kaolinite nanoroll (Kaol nanoroll), which was then added to PP together with IFR.^161^ The Kaol nanoroll showed a better synergistic effect on the thermal stability and fire resistance of PP than Kaol. In comparison to neat PP (1474 kW m^−2^), the pHRR values of PP/IFR (436 kW m^−2^), PP/IFR/1.5Kaol (372 kW m^−2^), and PP/IFR/1.5Kaol nanoroll (269 kW m^−2^) nanocomposites decreased by 70.3%, 74.4%, and 81.7%, respectively. According to the TGA data, the Kaol nanoroll may greatly increase the heat stability and char residues of the Kaol PP/IFR nanocomposites. Nanotubular HNT and nanoplate Kaol were introduced together in PP containing IFR.^162^ The composite containing 75% PP and 25% IFR had an LOI of 31.0% and a UL-94 grade of V-2, according to the data. Its LOI increased to 36.9% and the composite of 75 wt% PP, 23.5 wt% IFR, and 1.5 wt% (Kaol/HNT = 9/1) achieved a UL-94 grade of V-0; concurrently, its pHRR value in CC dropped by 82.2% in comparison to neat PP. Nano-roll and nano-sheet silicates were fabricated from natural kaolinite (Kaol). The two nano-sized silicates acted as synergists to enhance the fire-retardant performance of PP together with IFR. When 25 wt% IFR was added, the LOI increased to 31.1%. It then increased to 34.5% and 35.5% when 1.5 wt% of nano-rolled Kaol (N-Kaol) or exfoliated Kaol (E-Kaol) with the same amount of IFR was substituted, respectively.^163^ To enable PP to achieve a UL-94 V-0 grade, each modified Kaol can efficiently regulate melt dripping. Compared to the PP/IFR-containing E-Kaol, the macro/micro-structure of char and real-time FTIR showed that N-Kaol can help produce a homogenous and compact intumescent char layer through the “double effect” of barrier and pillar-like support during burning. Pentaerythritol phosphate was reacted with melamine and kaolin to obtain the melamine salt of pentaerythritol phosphate kaolin (MPPK).^164^ MPPK improved the thermal stability of PP. The vertical burning rate test manifested that PP composites can achieve V-0 at 20% and 25% MPPK loading levels. The data for PP containing 25% kaolin and 25% of conventional IFR made up of melamine phosphate (MP), pentaerythritol, and kaolin were compared with the results for the PP/25% MPPK composite. The results showed that MPPK outperformed the other systems in terms of flame retardancy. To further improve the flame retardancy and thermal stability characteristics of the PP-containing IFR composite, glycine (GLY) was intercalated and substituted into layers of kaolinite (Kaol-GLY).^165^ The LOI and char residues of the PP (75.0 wt%)/IFR (23.5 wt%) composite may increase in the presence of 1.5 wt% Kaol-GLY, whereas the peak heat and smoke release rate may decrease.

As a flame-retardant system in polyamide 6 (PA6), the interactions between kaolinite and a widely available phosphinate-based flame retardant (Exolit® OP1311) were assessed.^166^ Although OP1311 operates in the gas phase by inhibiting flames and diluting combustible gases (releasing ammonia) and in the condensed phase by promoting charring due to phosphinate, which may break down into phosphoric acid, kaolinite solely exhibits a condensed phase mode of action. The flame-prevention impact of OP may be limited by the ability of kaolinite to trap certain phosphorus and nitrogen compounds. It also likely enhances the barrier effect that shields the polymer matrix from radiant heat and slows down mass transfer from the gas phase.

Metal hydroxides, particularly those of aluminum and magnesium, provide the highest possibility to satisfy environmental regulations among flame retardants.^167^ However, they have poor mechanical properties given that significant loadings are required.^168,169^ At a lower global dosage, nanoparticles can also be employed in conjunction with standard flame retardants. The primary component of this type of mixture was organo-modified mineral nanoparticles. A previous study^170^ focused on the use of pristine and organo-modified Kaol to improve both the mechanical and flame retardancy properties of the PA6 material. The pHRR was reduced when a better dispersion of the biggest agglomerates was achieved.

By reacting 2,4-*N*-Tris(hydroxymethyl)methyl aminomethane (Tris), cyanuric chloride (CNC), and APP, a charring agent was created.^171^ To lessen the risk of fire and prevent smoke release, the resulting product Tris–CNC–APP was combined with a synergistic agent of MoS_2_@Acid-Kaol (molybdenum disulfide grafted on acidified kaolinite) in EP. Following the addition of 7% Tris–CNC–APP/3% MoS_2_@Acid-Kaol, the LOI of the EP sample was 36.1% and it achieved a UL-94 V-0 rating. Also, its THR and TSR were reduced by about half compared with the control EP sample. Ammonium dihydrogen phosphate (ADP) was intercalated into kaolinite to create intercalated kaolinite (K-ADP), which improved the thermal stability, flame retardance, and smoke suppression of EPs to increase the compatibility and flame retardance of Kaol in polymeric materials.^172^ The findings demonstrate that the addition of K-ADP has a greater beneficial impact on lowering the heat release and smoke production in EPs than the addition of Kaol. To improve the fire safety of EPs, K-ADP and APP were coupled.^173^ The flammability results show that compared to the combination of Kaol and APP, the combination of K-ADP and APP has extremely beneficial effects on lowering the heat release and smoke emission of EPs. When K-ADP and APP are combined, more phosphorus-rich crosslinking and aromatic structures can develop in the condensed phase, improving the compactness and intumescence of the char layer and effectively separating heat, combustible substances, and smoke particles. Molybdenum disulfide (MoS_2_) was grown on the surface of acidified kaolinite (A-Kaol) to produce MoS_2_@A-Kaol compounds, which improved the ageing resistance and thermal stability of EP, while successfully suppressing soot and harmful smoke.^174^ The TSP in the CC test was 3917.6 m^2^, but after adding 3 wt% MoS_2_@A-Kaol, the TSP value dropped to 2575.5 m^2^. MoS_2_@A-Kaol slowed down the mass loss and encouraged the char residue in the EP composite to increase to 11.3%. After the addition of 3 wt% MoS_2_@A-Kaol, the temperature for 50% mass loss in the TGA test was delayed from 388 °C to 414 °C, and the residue increased to 8.7%, suggesting that the thermal degradation of EP was delayed and its thermal stability was enhanced.

In the case of the PVC/kaolinite composite, the measurement results showed a significant increase in burning duration and a significant drop in burning rate; in other words, the higher the kaolin content, the longer the burning time and the slower the burning rate.^175^ The effects of kaolinite particle size and kaolinite content on the thermal stability of a kaolinite/styrene butadiene rubber (SBR) composite was investigated.^176^ Compared to pure SBR, the produced composite showed a notable improvement in thermal stability. As the particle size decreased and the concentration increased, the typical breakdown temperatures of the kaolinite/SBR composite gradually increased. The fine dispersion of kaolinite particles in the rubber matrix and the robust interactions between kaolinite particles and rubber chains are responsible for the improved thermal stability of the kaolinite/SBR composite. To enhance the flame retardancy and smoke suppression of EVA composites containing IFR, kaolinite (AKaol) pre-acidified with sulfuric acid was subsequently treated by loading TiO_2_ and doping Zn^2+^ to create Zn/TiO_2_@AKaol.^177^ The findings demonstrated that the addition of 1 wt% Zn/TiO_2_@AKaol enhanced the flame retardancy of the EVA/IFR composite and decreased its smoke release. The LOI value of the Zn/TiO_2_@AKaol-containing EVA/IFR composite increased from 27.3% to 32.7%. To achieve flame retardancy and smoke suppression in EVA composites, it was suggested based on the mechanism analysis that Zn/TiO_2_@AKaol increased the formation of char with a cross-linked structure in the condensed phase, in addition to encouraging the formation of P˙ in advance. To increase the flame retardancy of EVA composites, a modified kaolinite created by grafting phytic acid (PA-g-Kaol) was added to EVA together with IFR.^178^ The findings show that some pyrolytic products of IFR and PA-g-Kaol, such as phosphoric acid and ammonia, catalyze the crosslinking of EVA and the flame retardant; the resulting compact char shields the substrate from additional burning. To improve the thermal stability and flame retardancy of intumescent flame-retarding polyurea (PUA/IFR) composites, kaolinite was added.^179^ The findings indicate that the IFR system and kaolinite have a synergistic impact, resulting in excellent flame retardance and outstanding fire protection. The LOI value of the PUA/IFR composite increased to 36.0% when 1.0 wt% kaolinite was added (along with the remaining 24% IFR), and the time to flame out and THR significantly decreased by 32.0% and 16.0%, respectively.

Table 4 presents some results on the influence of kaolinite on the flammability of polymer composites.

**Table 4:** Fire properties of kaolinite-based polymer composites

Formulation of composites		LOI	UL-94	PHRR (kW m^−2^)	Reference
Matrix	Additive				
LDPE	SiO_2_@MAPP(10.83 g) + DPER(5.42 g)	24.1	No	—	155
	SiO_2_@MAPP(10.63 g) + DPER(5.32 g) + KU(0.30 g)	24.7	No		
	SiO_2_@MAPP(10.23 g) + DPER(5.12 g) + KU(0.90 g)	27.2	V-1		
	SiO_2_@MAPP(9.83 g) + DPER(4.92 g) + KU(1.50 g)	26.5	V-1		
PBS	—	21.9	NR	576.3	156
	25IFR	31.7	NR	445.5	
	20IFR + 5Kaol	38.3	V-0	305.1	
	20IFR + 5K-U	40.1	V-0	292.4	
PP	—	18.0 ± 0.2	NR	1474	157
				—	
	25IFR	31.1 ± 0.2	V-2	329	
				438	
	23.5IFR + 1.5Koal	32.5 ± 0.2	V-0	326	
				373	
	22IFR + 1.5Acid-Koal	34.9 ± 0.2	V-0	186	
				233	
PP	—	18.0 ± 0.1	NR	1474	158
	IFR	31.1 ± 0.2	V-2	329	
				438	
	1.5K_0_	18.3 ± 0.1	NR	1346	
	IFR + 1.5K_0_	32.5 ± 0.2	V-0	326	
				373	
	1.5AS-K	18.6 ± 0.2	NR	1169	
	IFR + 1.5AS-K	35.3 ± 0.2	V-0	316	
				309	
PP	—	18.0 ± 0.1	NR	1474 ± 28	161
	IFR	31.1 ± 0.2	V-2	329 ± 2 0	
				438 ± 20	
	IFR + 1.5Kaol	32.5 ± 0.2	V-0	326 ± 17	
				373 ± 17	
	IFR + 1.5Kaol nanoroll	34.5 ± 0.2	V-0	252 ± 14	
				269 ± 14	
PP	—	18.0 ± 0.1	NR	1474 ± 28	162
	25IFR	31.0 ± 0.1	V-2	438 ± 20	
	23.5IFR + 1.5Kaol	32.5 ± 0.2	V-0	373 ± 17	
	23.5IFR + 1.5HNT	35.2 ± 0.2	V-0	341 ± 15	
	23.5IFR + 1.5(9Kaol:1HNT)	36.9 ± 0.2	V-0	236 ± 14	
PP	—	18.0 ± 0.1	NR	1474 ± 28	163
	IFR	31.1 ± 0.2	V-2	329 ± 20	
				438 ± 20	
	IFR + 1.5Kaol	18.3 ± 0.1	NR	326 ± 17	
				373 ± 17	
	IFR + 1.5E-Kaol	35.5 ± 0.2	V-0	236 ± 14	
				319 ± 15	
	IFR + 1.5N-Kaol	34.5 ± 0.2	V-0	252 ± 14	
				269 ± 14	
PP	—	17.4 ± 0.1	NR	1547 ± 26	165
	25IFR	27.3 ± 0.2	NR	496 ± 14	
	23.5IFR + 1.5Kaol	29.8 ± 0.2	NR	367 ± 10	
	23.5IFR + 1.5Kaol-GLY	32.9 ± 0.2	V-0	317 ± 8	
EVA	—	20.9 ± 0.1	NR	865 ± 25.3	178
	20IFR	24.9 ± 0.2	V-2	382.1 ± 18.4	
				359.5 ± 20.7	
	18IFR + 2Kaol	28.6 ± 0.2	V-2	—	
	18IFR + 2Pa-g-Kaol	30.8 ± 0.2	V-2	—	
PUA	—	22.4 ± 0.1	V-2	578 ± 22	179
				—	
	20APP + 5CFA	35.0 ± 0.1	V-0	160 ± 12	
				230 ± 32	
	19.6APP + 4.9CFA + 0.5K_0_	35.2 ± 0.2	V-0	109 ± 20	
				234 ± 32	
	19.2APP + 4.8CFA + 1K_0_	36.0 ± 0.2	V-0	128 ± 18	
				235 ± 17	
	18.4APP + 4.6CFA + 2K_0_	35.5 ± 0.1	V-0	159 ± 22	
				258 ± 35	

### 3.12. Flame-retardant polymer composites based on wollastonite

Historically, 3 essential viewpoints were considered when evaluating the suitability of a chemical as a flame retardant, including the type and quantity of harmful gases emitted, the degree to which its mechanical characteristics are compromised, and its impact on hygroscopic behavior.^180^ Although all these aspects have been discussed in detail over the years, only recently environmental and health issues have gained attention. In this context, wollastonite (Wo) has been shown to present no health risks to humans or wildlife; it is a silicate compound (CaSiO_3_) that is free from chemical pollution and does not adversely affect mechanical properties given that it lacks any acidic substances (Table 1). Wo is a calcium-rich silicate and a mineral that forms rocks in the upper crust.^181^ It exists in two polymorphic forms, α- and β-Wo, and each polymorph is characterized by several polytypes. β-Wo, which is commonly referred to as Wo, is classified as a chain-silicate and features a structure composed of infinitely long single chains. These chains are composed of a couple of two-silicon tetrahedra, Si_2_O_7_, paired to one silicon tetrahedron, SiO_4_.

Flame-retardant EP/cyanate ester composites were obtained using DOPO and Wo.^182^ It was found that the EP/cyanate ester composite containing 7 wt% DOPO and 3 wt% Wo exhibited the best flame retardancy (LOI 35.5% and UL-94 V-0 rating). IFR coatings were obtained to study the combined effects of reinforced mica and Wo-based IFR coatings concerning heat shielding, char expansion and morphology.^183^ APP was used as an acid source, EG as a carbon source, melamine as a blowing agent, boric acid as an additive and Hardener H-2310 polyamide amine in bisphenol A epoxy resin BE-188(BPA) as a curing agent.

Wo was used together with IFR to prepare flame-retardant PP.^184^ The results revealed that wollastonite could effectively improve the mechanical properties and flame retardancy of the PP/IFR composite. The LOI increased from 33.0% to 35.5%, a UL-94 V-0 rating was achieved and the pHRR significantly dropped from 314.4 kW m^−2^ to 262.8 kW m^−2^. Additionally, the SEM–EDS findings confirmed that the quality of the char layer in the PP/IFR/Wo composite was superior to that of the PP/IFR composite as a result of the synergistic interaction between Wo and IFR. Synthetic Wo nanofibers were incorporated into polybutylene terephthalate (PBT) to study the effect of Wo content on flammability properties.^185^ Wo improved the flame retardancy by reducing the pHRR by about 17% and the production of smoke and toxic gases by 23–36%.

## 4. Mechanical properties of mineral filler-based fire-resistant polymer composites

Although the use of inorganic minerals as flame retardants is a much safer alternative to halogen-containing ones, it is seen that while they impart non-flammability, they impair some mechanical properties of polymer composites. Attaining a satisfactory mechanical performance in a polymer composite is a key priority when utilizing mineral-based flame retardants. To ensure a significant degree of flame resistance, these mineral-based flame retardants should be incorporated into the matrix at levels ranging from 50% to 70%.^44^ A high concentration of minerals enhances the modulus of elasticity, while diminishing the ductility of the material. This could pose challenges in scenarios where flexibility is essential. To address this issue, minerals can be combined with other efficient flame retardants.

According to the data obtained,^186^ samples containing huntite–hydromagnesite, which demonstrated a flame-retardant UL94 V0 rating, showed superior mechanical properties, with tensile strength ranging from 23.4% to 70.8%, and elongation at break ranging from 60.7% to 73.1%. In a previous study,^45^ calcite and zeolite minerals, with well-known incombustibility properties, were used together with huntite hydromagnesite as auxiliary minerals to obtain better mechanical properties. By loading zeolite together with huntite hydromagnesite, the maximum tensile strength increased from 15.6 MPa to 22.9 MPa, resulting in an increase of 46.80%. Research revealed that^187^ incorporating the mineral HH led to a decline in the mechanical strength of the polymer–wood–mineral composite owing to the increase in its brittleness. In terms of balance between mechanical and fire resistance, the optimal polymer–wood–mineral sample had a mineral content of 20–30%. The synergistic effect of ZnB on the flame-retardant and mechanical and thermal properties of huntite/hydromagnesite-reinforced PP composites was investigated.^38^ The elastic modulus of PP was measured to be 1000 MPa, and the maximum loading of 50 wt% increased its elastic modulus to 2.3 GPa.

Sepiolite shows remarkable characteristics over various fillers due to its higher specific surface area and channel-type structure. It exhibited better hardness, flexural, tensile, and impact properties compared with powder-fortified polymer composites.^188^ Apart from decreasing the burning rate up to 48%, thermal stability and tailored mechanical properties are also achieved.^55^ As the content of organically modified sepiolites increases, the flowability of PP/organically modified sepiolite composites is initially enhanced due to the lubricating effect of surface modifiers, before diminishing because of the interactions between the organically modified sepiolites and PP.^189^ The addition of 4 wt% sepiolite resulted in a 15% boost in the tensile strength and a 25% increase in the Young's modulus of PS nanocomposites.^190^

The incorporation of a buckwheat hull/perlite combination was found to influence the physical–mechanical characteristics of prepared rigid polyurethane foams, such as apparent density, impact strength, and compressive and flexural strength.^130^ The testing results showed that the flexural modulus of a composite increased by approximately 37.5% with the addition of perlite.^191^

It was observed that the tensile strength and elongation at break of PVC composites decreased as the kaolinite content increased.^192^ Furthermore, the hardness of these composites increases with an elevation in filler quantity. Rheological measurements showed that surface treatment of kaolinite leads to a decrease in its viscosity, which could enhance its processability.^160^ Mechanical testing indicates that the incorporation of K-ADP exerts a less detrimental impact on the mechanical properties of EP composites compared to Kaol, which is attributed to the superior compatibility of ammonium dihydrogen phosphate-intercalated kaolinite with the epoxy matrix.^173^

Both the mechanical and thermal properties of polymers were remarkably improved when mixed with HNTs.^193^ Owing to its relatively high aspect ratio and hardness, wollastonite enhances the tensile and flexural strength.^194^ Some indicators reflecting mechanical properties are given in Table 5.

**Table 5:** Indicators reflecting the mechanical properties of composites

Formulation of composites		Tensile strength, MPa	Elongation at break, %	Reference
Matrix	Additive			
ABS	—	44.3 ± 2.0	16.0 ± 1.2	36
	25% AlNP	37.4 ± 1.5	13.4 ± 0.4	
	22% AlNP + 3% 4A zeolite	36.4 ± 1.2	11.8 ± 0.3	
TPU	—	24.8 ± 2.2	424 ± 22.0	47
	50HH	9.3 ± 0.8	21 ± 1.3	
	43HH + 7mRP	15.0 ± 0.3	148 ± 10	
TPU	—	24.8 ± 2.2	424.0 ± 22.3	49
	25HH + 25EG	9.6 ± 0.3	44.6 ± 1.5	
PE	HH	11.0	93	51
EVA + PE	HH	12.7	144	
PP	—	32.7 ± 0.6	133.8 ± 2.9	101
	15% PN-DOPO + 5% OMMT	44.9 ± 1.2	112.8 ± 3.4	
EVA	—	11.59 ± 0.14	735.51 ± 8.27	117
	19.5 g APP + 6.5 g TPU + 3 g OMMT + 1 g GNSs	8.56 ± 0.28	650 ± 12.61	
PS	—	49.4	9.4	123
	5% MMT	37.0	7.1	
	5% MMT + 1% ZnO	49.5	8.6	
	5% MMT + 5% ZnO	44.0	7.6	
PVC	—	12.43	580.98	126
	40 g dioctylphthalate + 0.5 g lead sulfate tribasic + 0.5 g dibasic lead phosphate + 6.5 g MMT	12.23	584.33	
	40 g dioctylphthalate + 0.5 g lead sulfate tribasic + 0.5 g dibasic lead phosphate + 6.5 gMMT-Fe_2_O_3_	12.42	559.27	
LDPE	10.83 g SiO_2_@MAPP + 5.42 g DPER	16.1 ± 0.6	512 ± 7.2	155
	10.63 g SiO_2_@MAPP + 5.32 g DPER + 0.3 g KU	15.7 ± 0.3	507 ± 9.8	
	10.23 g SiO_2_@MAPP + 5.12 g DPER + 0.9 g KU	16.6 ± 1.2	554 ± 7.4	
	9.83 g SiO_2_@MAPP + 4.92 g DPER + 1.50 gKU	17.8 ± 0.2	561 ± 5.6	

## 5. Conclusion and prospect

This article provides a current review of flame-retardant polymer composites based on mineral fillers. The flame-retardant performances of mineral filler-based composites are discussed according to the type of mineral filler and nature of the polymer matrix. Researchers have invested significant effort into developing environmentally friendly and flame-retardant materials, which are detailed herein. Given that most mineral fillers have poor fire protection properties, they must be combined with other flame retardants to increase their functionality. Nevertheless, the development of flame-retardant polymer materials based on mineral fillers still faces many challenges. The following observations were made from this review: mineral flame retardants often work through more complex mechanisms (such as endothermic decomposition and heat capacity increase), which might make it harder to optimize their performance.

The development of flame-retardant polymer materials based on mineral fillers is a promising and sustainable direction for their real industrial application: many mineral fillers have natural origins and can be considered more environmentally friendly compared to chemical flame retardants; mineral fillers are often a cost-effective and readily available, making them more practical and effective to use; some mineral fillers can help suppress the release of smoke and toxic gases during combustion, helping to create a safer fire environment; mineral fillers can be incorporated into a wide range of polymers; and some mineral fillers provide good thermal stability and can help prevent premature polymer degradation.

## Author contributions

Fatima Mustafayeva: writing – review & editing, writing – original draft, concepualization, data curation. Najaf Kakhramanov: writing – review & editing. Khayala Allahverdiyeva: visualization. Tunzala Babayeva: visualization. Nargiz Ashurova: visualization.

## Conflicts of interest

The authors declare that they have no known competing financial interests or personal relationships that could have appeared to influence the work reported in this paper.

## Abbreviations

ABS: Acrylonitrile–butadiene–styrene copolymer
AlNP: Aluminum β-(*p*-nitrobenzamide)ethyl methyl phosphinate
APP: Ammonium polyphosphate
ATH: Aluminum hydroxide
BF: Basalt fiber
BR: Burning rate
CC: Cone calorimeter
CFA: Charring-forming agent
EG: Expandable graphite
EP: Epoxy resin
EV: Expanded vermiculite
EVA: Ethylene-vinyl acetate copolymer
HDPE: High-density polyethylene
HH: Huntite & hydromagnesite
HNT: Halloysite nanotubes
HRR: Heat release rate
IFR: Intumescent flame retardant
LDPE: Low-density polyethylene
LLDPE: Linear low-density polyethylene
LOI: Limiting oxygen index
MLR: Mass loss rate
NR: Not rated
OMMT: Organic montmorillonite
pHRR: Peak heat release rate
PP: Polypropylene
PS: Polystyrene
PU: Polyurethane
PVC: Polyvinyl chloride
TFO: Time to flame out
TGA: Thermogravimetric analysis
THEIC: Tris(2-hydroxyethyl)isocyanurate
THR: Total heat release
TPU: Thermoplastic polyurethane
TSP: Total smoke production
TSR: Total smoke released
TTI: Time to ignition
VMT: Vermiculite
Wo: Wollastonite
ZnB: Zinc borate

## Data Availability

All data are obtained from peer-reviewed articles as reported in the References list. No other datasets have been used.
